# Systemic silencing of an endogenous plant gene by two classes of mobile 21‐nucleotide artificial small RNAs

**DOI:** 10.1111/tpj.15730

**Published:** 2022-03-27

**Authors:** Adriana E. Cisneros, Ainhoa de la Torre‐Montaña, Alberto Carbonell

**Affiliations:** ^1^ Instituto de Biología Molecular y Celular de Plantas (Consejo Superior de Investigaciones Científicas – Universitat Politècnica de València) 46022 Valencia Spain

**Keywords:** systemic silencing, small RNA movement, cell non‐autonomous, artificial microRNA, synthetic *trans*‐acting siRNA, transitivity, *Nicotiana benthamiana*

## Abstract

Artificial small RNAs (art‐sRNAs) are 21‐nucleotide small RNAs (sRNAs) computationally designed to silence plant genes or pathogenic RNAs with high efficacy and specificity. They are typically produced in transgenic plants to induce silencing at the whole‐organism level, although their expression in selected tissues for inactivating genes in distal tissues has not been reported. Here, art‐sRNAs designed against the magnesium chelatase subunit CHLI‐encoding *SULFUR* gene (*NbSu*) were agroinfiltrated in *Nicotiana benthamiana* leaves, and the induction of local and systemic silencing was analyzed phenotypically by monitoring the appearance of the characteristic bleached phenotype, as well as molecularly by analyzing art‐sRNA processing, accumulation and targeting activity and efficacy. We found that the two classes of art‐sRNAs, artificial microRNAs (amiRNAs) and synthetic *trans*‐acting small interfering RNAs (syn‐tasiRNAs), are able to induce systemic silencing of *NbSu*, which requires high art‐sRNA expression in the vicinity of the leaf petiole but is independent on the production of secondary sRNAs from *NbSu* mRNAs. Moreover, we revealed that 21‐nucleotide amiRNA and syn‐tasiRNA duplexes, and not their precursors, are the molecules moving between cells and through the phloem to systemically silence *NbSu* in upper leaves. In sum, our results indicate that 21‐nucleotide art‐sRNAs can move throughout the plant to silence plant genes in tissues different from where they are produced. This highlights the biotechnological potential of art‐sRNAs, which might be applied locally for triggering whole‐plant and highly specific silencing to regulate gene expression or induce resistance against pathogenic RNAs in next‐generation crops. The present study demonstrates that artificial small RNAs, such as artificial microRNAs and synthetic *trans*‐acting small interfering RNAs, can move long distances in plants as 21‐nucleotide duplexes, specifically silencing endogenous genes in tissues different from where they are applied. This highlights the biotechnological potential of artificial small RNAs, which might be applied locally for triggering whole‐plant, highly specific silencing to regulate gene expression or induce resistance against pathogenic RNAs in next‐generation crops.

## Significance Statement

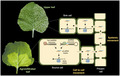

## INTRODUCTION

Eukaryotic small RNAs (sRNAs) are responsible for the sequence‐specific degradation of highly sequence complementary RNA molecules, a process known as RNA silencing. In plants, sRNAs arise from the processing of a double‐stranded RNA (dsRNA) by DICER‐LIKE (DCL) enzymes into 21–24‐nucleotide duplexes. Typically, one of the strands of the duplex (the guide strand) is incorporated into an ARGONAUTE (AGO) protein that recognizes and inactivates complementary target RNA by diverse mechanisms (Axtell, 2013; Bologna & Voinnet, 2014; Borges & Martienssen, 2015). In some contexts, silencing can be amplified through the production of secondary sRNAs by RNA‐DEPENDENT RNA POLYMERASES (RDRs); for example, upon the targeting of RNAs by 22‐nucleotide sRNAs (Chen et al., 2010; Cuperus et al., 2010), a process termed transitivity. RNA silencing can also function non‐cell autonomously in plants because sRNAs can spread from cell to cell, over long‐distances and even between different plants or interacting organisms ( Dunker et al., 2020; Liu & Chen, 2018; Weiberg et al., 2015). For example, sRNA short‐range movement extends 10–15 cells away from the production site and occurs via plasmodesmata connecting the cytoplasm of adjacent cells, whereas long‐distance traffic to distal tissues occurs through the phloem (Molnar et al., 2011).

Artificial sRNAs (art‐sRNAs), such as artificial microRNAs (amiRNAs) and synthetic *trans*‐acting small interfering RNAs (syn‐tasiRNAs) are 21‐nucleotide sRNAs computationally designed to selectively silence one or more target genes with high specificity and efficacy (Carbonell, 2017, 2019; Ossowski et al., 2008; Tiwari et al., 2014; Zhang, 2014). The amiRNAs are typically produced *in planta* by expressing an endogenous miRNA precursor in which the sequences of the endogenous miRNA/miRNA* are replaced by the amiRNA/amiRNA* sequences. The amiRNA primary transcript is sequentially processed by DCL1 to release the 21‐nucleotide amiRNA duplex. Syn‐tasiRNAs are produced by expressing an endogenous *TAS* precursor including one or more syn‐tasiRNA sequences replacing endogenous tasiRNA sequences. In this case, the syn‐tasiRNA primary transcript is cleaved by a miRNA/AGO complex, RDR6‐including complexes produce a dsRNA from one of the *TAS* cleaved fragments, and the resulting dsRNA is sequentially processed by DCL4 in 21‐nucleotide syn‐tasiRNA duplexes in phase with the miRNA trigger target site. For both art‐sRNA classes, the guide strand of the art‐sRNA duplex (typically including a 5′ U) associates with AGO1 to silence complementary RNA(s). The art‐sRNAs are extensively used in plants to regulate gene expression in gene function studies and to induce antiviral resistance (Cisneros & Carbonell, 2020; Cisneros et al., 2021)]. However, one major limitation of current art‐sRNA approaches is that art‐sRNA are usually expressed in transgenic organisms, which hinders the commercialization of art‐sRNA‐expressing crops in regions where genetically modified organisms (GMOs) are banned. In this context, the possibility of expressing art‐sRNAs in one tissue for triggering the systemic silencing (SS) of a target RNA in a different tissue should facilitate the control of gene expression and/or induce antiviral resistance at the whole‐plant level and in line with international laws. Unfortunately, the molecular bases governing the induction and spread of SS are not always clear, particularly for endogenes, which has hampered the development of efficient methods for triggering SS in plants.

Here, we report that the transient expression in *Nicotiana benthamiana* leaves of amiR‐NbSu‐2, a 21‐nucleotide amiRNA designed to silence the magnesium chelatase subunit CHLI‐encoding *SULFUR* gene (*NbSu*), induces obvious SS in upper non‐agroinfiltrated leaves. SS was characterized by a strong near‐vein chlorosis and reduced *NbSu* mRNA levels, and was triggered only when amiR‐NbSu‐2 was highly expressed in tissues neighboring the petiole. Interestingly, the expression of a 22‐nucleotide form of amiR‐NbSu‐2 did not induce SS, despite triggering the biogenesis of 21‐nucleotide phased secondary small interfering RNAs (siRNAs) from *NbSu* mRNAs. By combining sRNA high‐throughput sequencing, 5′‐RNA ligase‐mediated rapid amplification of cDNA ends (RLM‐RACE) and reverse transcriptase‐polymerase chain reaction (RT‐PCR) analyses, we show that amiR‐NbSu‐2 is present and active in systemically silenced tissues, where it specifically cleaves *NbSu* mRNAs. Moreover, the transient expression of syn‐tasiR‐NbSu‐2, a 21‐nucleotide syn‐tasiRNA of identical sequence to amiR‐NbSu‐2, also triggers SS in a similar way. Our results indicate that both amiRNAs and syn‐tasiRNAs can trigger the silencing of a plant endogenous gene in a distal tissue from where they are produced, in a process not requiring transitivity. They also suggest that both classes of art‐sRNAs are able to move as 21‐nucleotide sRNA duplexes from cell to cell via plasmodesmata and long‐distances to sink tissues through the phloem.

## RESULTS

### Local silencing of *N. benthamiana magnesium chelatase subunit CHLI* (*NbSu*) by specific amiRNAs causes strong bleaching phenotype in amiRNA‐expressing tissues

**Figure 1:**
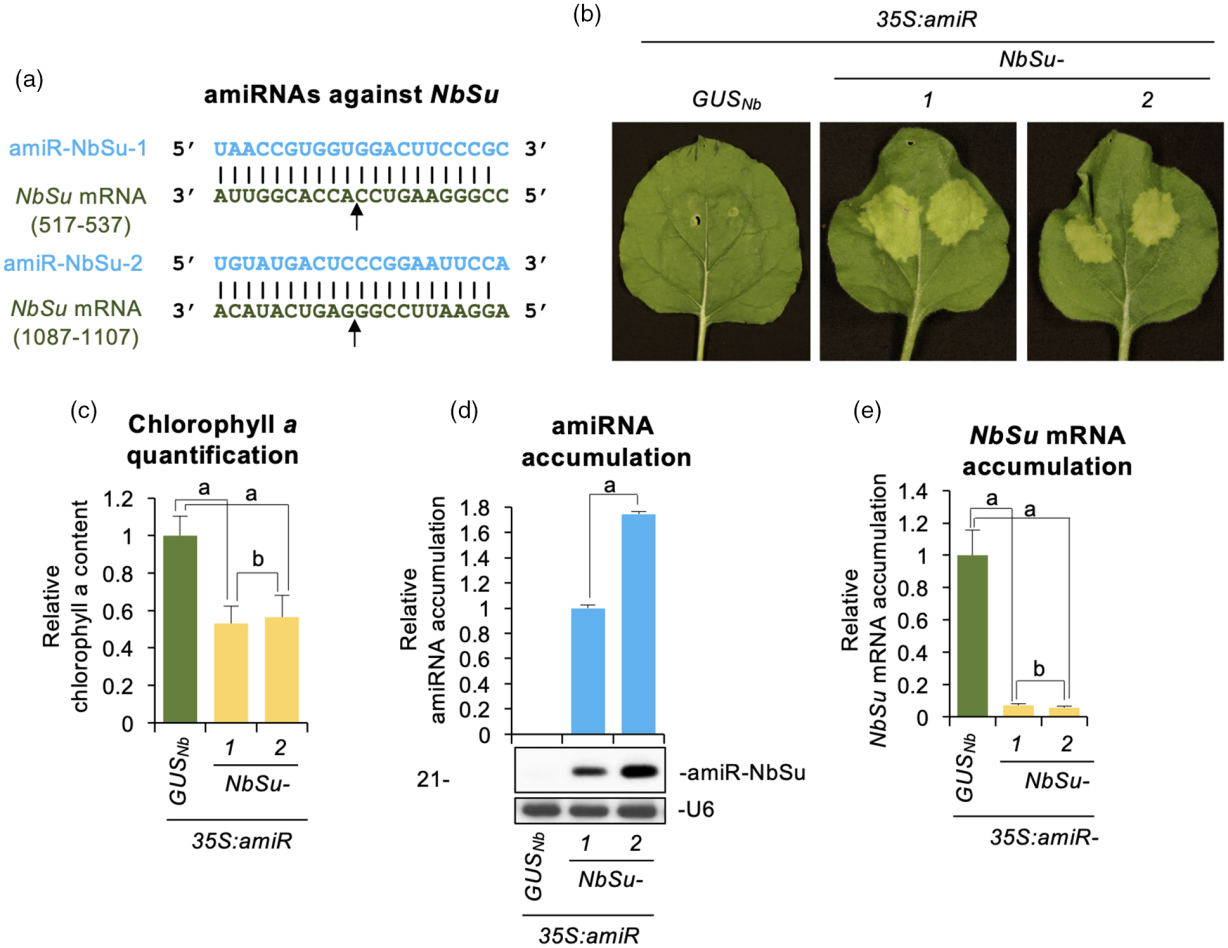
Functional analysis of artificial microRNAs (amiRNAs) against *N. benthamiana SULFUR* (amiR‐NbSu) in agroinfiltrated leaves. (a) Base‐pairing of amiRNAs and *NbSu* target mRNAs. Coordinates of the complete target site in *NbSu* mRNAs are given. The arrows indicate the amiRNA‐predicted cleavage site. (b) Photographs at 7 days post‐agroinfiltration (dpa) of leaves agroinfiltrated with the different amiRNA constructs. (c) Bar graph showing the relative content of chlorophyll *a* in agroinfiltrated areas (*35S:amiR‐GUS*_*Nb*_ = 1.0). Bars with the letter ‘a’ are significantly different from that of sample *35S:amiR‐GUS*_*Nb*_ (*P* < 0.01 in pairwise Student's *t*‐test comparisons). (d) Northern blot detection of amiR‐NbSu amiRNAs in RNA preparations from agroinfiltrated leaves at 2 dpa. The graph at top shows the mean ± SD (*n* = 3) amiR‐NbSu relative accumulation (*35S:amiR‐NbSu‐1* = 1.0). The bar with the letter ‘a’ is significantly different from that of sample *35S:amiR‐NbSu‐1*. Other details are as shown in (c). (e) Accumulation of *NbSu* mRNA. Mean mean ± SE relative level (*n* = 3) of *NbSu* mRNAs after normalization to *PROTEIN PHOSPHATASE 2A* (*PP2A*), as determined by quantitative RT‐PCR (qPCR) (*35S:amiR‐GUS*_*Nb*_ = 1.0 in all comparisons). Other details are as shown in (b). [Colour figure can be viewed at wileyonlinelibrary.com]

Two amiRNA constructs (*35S,amiR‐NbSu‐1* and *35S:amiR‐NbSu‐*2), each expressing an amiRNA with no predicted off‐targets in *N. benthamiana* and targeting a unique site in *NbSu* mRNA, were generated and independently agroinfiltrated in two areas of two leaves from three different *N. benthamiana* plants (Figure 1a). As a negative control, the *35S:amiR‐GUS*_*Nb*_ construct expressing a highly specific amiRNA against *Escherichia coli* β‐glucuronidase uida gene was also generated and agroinfiltrated. At 7 days post‐agroinfiltration (dpa), areas agroinfiltrated with anti‐*NbSu* amiRNA constructs displayed a visually obvious bleached phenotype characteristic of *NbSu* knockdown (Tang et al., 2010), whereas areas expressing *35S:amiR‐GUS*_*Nb*_ did not (Figure 1b). The chlorophyll analysis of agroinfiltrated areas showed a similar decrease in chlorophyll content in bleached areas expressing anti‐*NbSu* amiRNA constructs compared to areas expressing the control construct (Figure 1c). To molecularly characterize amiRNA activity, two leaves of each of three different *N. benthamiana* plants were independently agroinfiltrated in the whole leaf surface with the amiRNA constructs described above, and amiRNA and target mRNA accumulation were analyzed at 2 dpa. RNA blot assays of RNA preparations from agroinfiltrated leaves showed that both anti‐*NbSu* amiRNAs accumulated as single‐size sRNA species, with amiR‐NbSu‐2 accumulating to significantly higher levels than amiR‐NbSu‐1 (Figure 1d). Finally, quantitative RT‐PCR (RT‐qPCR) analysis revealed that samples expressing anti‐*NbSu* amiRNAs accumulated significantly lower levels of *NbSu* mRNA than control samples (Figure 1e). Taken together, these results indicate that both anti‐*NbSu* amiRNAs are highly active and induce a strong bleaching phenotype in the tissue where they are expressed.

### Systemic silencing of *NbSu* in distal tissues is induced by the agroinfiltration of one of the two anti‐ *NbSu* amiRNA

**Figure 2:**
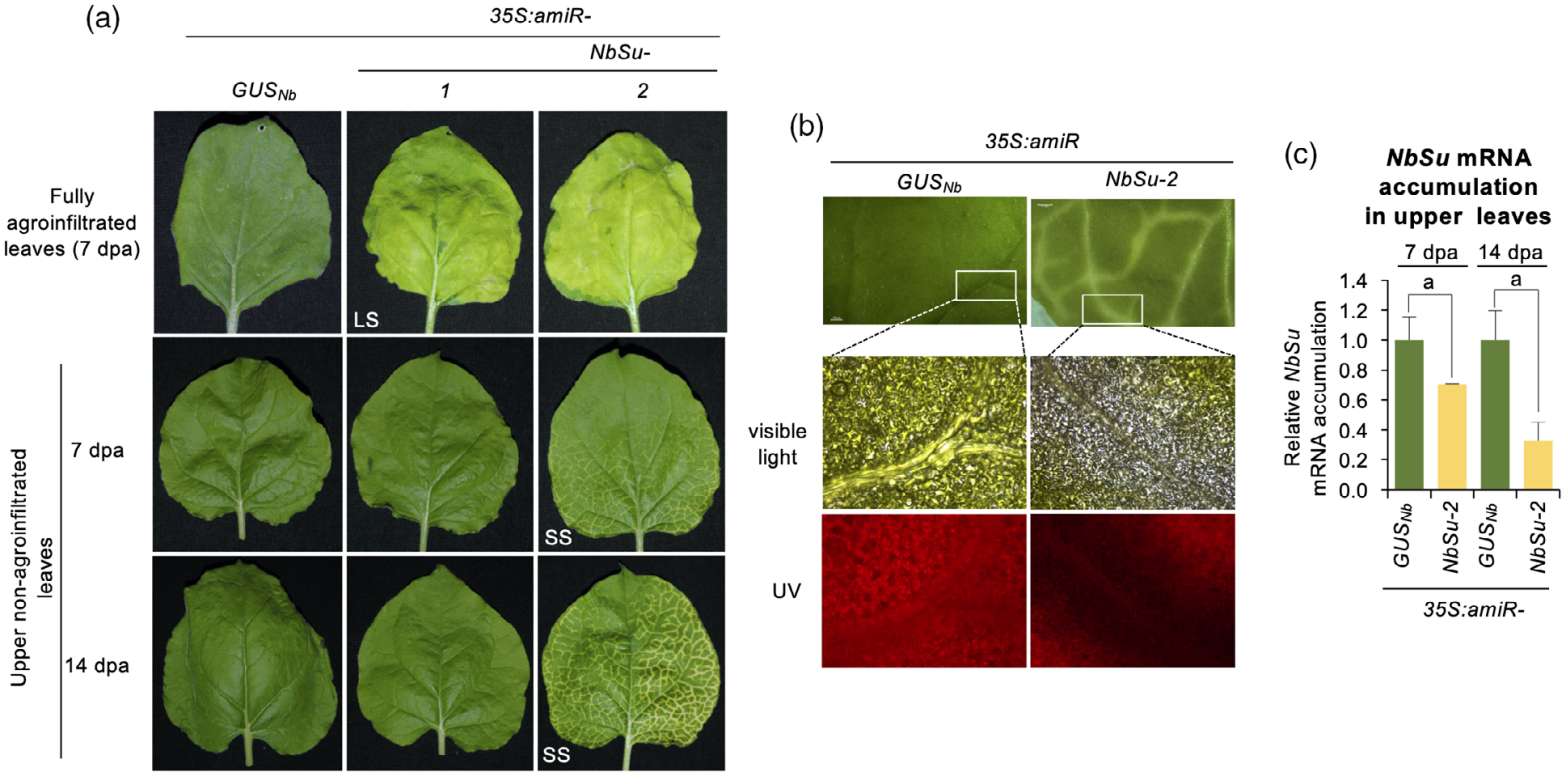
Analysis of upper non‐agroinfiltrated tissues showing systemic silencing. (a) Photographs of fully agroinfiltrated leaves at 7 days post‐agroinfiltration (dpa) (top) and of upper non‐agroinfiltrated leaves at 7 dpa (middle) and 14 dpa (bottom). LS and SS refer to local and systemic silencing, respectively. A white arrow point to areas displaying near‐vein chlorosis. (b) Photographs of areas surrounding leaf nerves under visible or ultraviolet (UV) light from plants agroinfiltrated with *35S:amiR‐GUS*_*Nb*_ or *35S:amiR‐NbSu‐2*. (c) Accumulation of *NbSu* mRNA at 7 and 14 dpa in upper leaves of plants agroinfiltrated with *35S:amiR‐GUS*_*Nb*_ or *35S:amiR‐NbSu‐2*. Mean ± SE relative level (*n* = 3) of *NbSu* mRNAs after normalization to *PROTEIN PHOSPHATASE 2A* (*PP2A*), as determined by quantitative RT‐PCR (qPCR) (*35S:amiR‐GUS*_*Nb*_ [7 dpa] = 1.0 in all comparisons). Other details are as shown in Figure 1(b). [Colour figure can be viewed at wileyonlinelibrary.com]

Remarkably, when amiRNA constructs were agroinfiltrated in the whole surface of *N. benthamiana* leaves, a bleaching phenotype was also observed in upper non‐agroinfiltrated leaves as early as 3 dpa only in plants agroinfiltrated with *35S:amiR‐NbSu‐2*. The distal bleaching phenotype was characterized by a vein‐proximal chlorosis and was more intense at 7 dpa and even more at 14 dpa (Figure 2a). A closer analysis of chlorophyll autofluorescence confirmed that the bleached areas extended beyond the veins (Figure 2b), most likely to 10–15 cells as reported for siRNAs (Himber et al., 2003). Importantly, RT‐qPCR analysis showed that *NbSu* mRNA accumulation in distal silenced tissues was significantly decreased in plants expressing *35S:amiR‐NbSu‐*2 compared to plants expressing *35S:amiR‐GUS*_*Nb*_ (Figure 2c). Moreover, this decrease was correlated with the bleaching intensity because *NbSu* mRNA levels were significantly lower at 14 dpa compared to 7 dpa (Figure 2c). In sum, these results suggest that a silencing signal moves from source agroinfiltrated tissues to upper parts of the plants.

### Systemic silencing requires amiRNA expression near the petiole and positively correlates with the level of amiRNA accumulation

**Figure 3:**
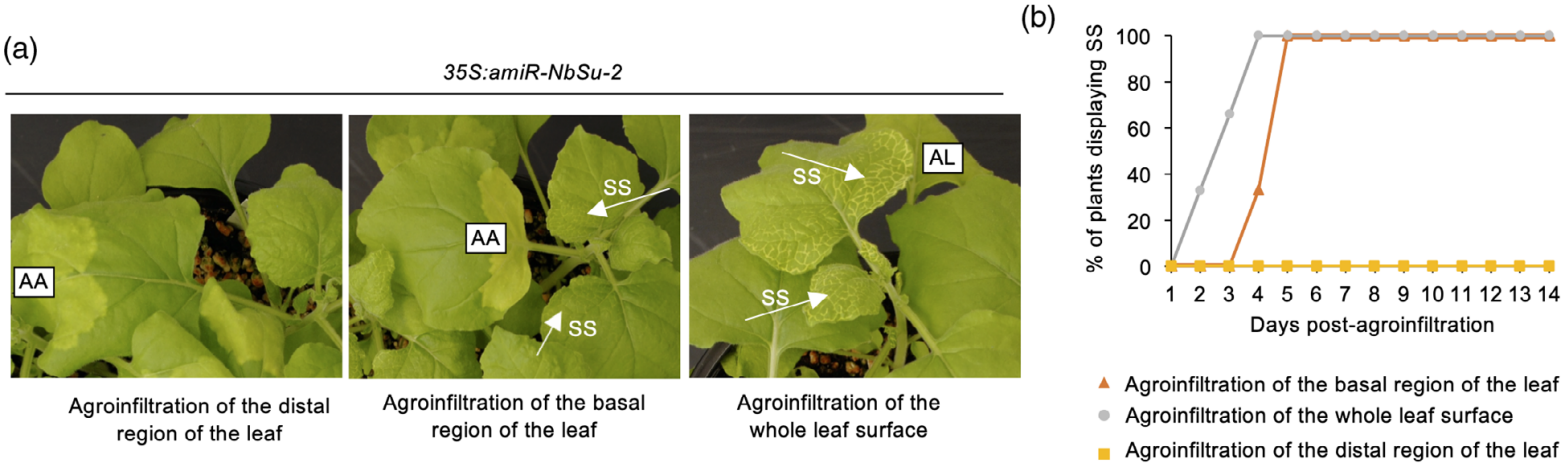
Effects on systemic silencing of the place of expression of amiR‐NbSu‐2 in *N. benthamiana* leaves. (a) Photographs of plants at 7 days post‐agroinfiltration (dpa). AA and AL refer to agroinfiltrated area and agroinfiltrated leaf, respectively. Near‐vein chlorotic areas result of systemic silencing (SS) are indictated with a white arrow. (b) Two‐dimensional line graph showing, for each of the three‐plant sets listed in the box, the percentage of plants displaying systemic silencing per day during 14 dpa. [Colour figure can be viewed at wileyonlinelibrary.com]

Our previous experiment revealed that amiR‐NbSu‐2 triggered SS only when expressed in the whole leaf surface, but not when the amiRNA was expressed in a distal region of the leaf. Thus, we hypothesized that the movement of the silencing signal might require the expression of amiR‐NbSu‐2 in tissues surrounding the petiole connecting the leaf and stem vasculatures. To confirm this, *35S:amiR‐NbSu‐2* was independently agroinfiltrated in the basal region of one leaf of three different plants. The same construct was independently agroinfiltrated in the whole leaf surface or in a distal region of a leaf of three different plants as positive and negative control, respectively. The presence of local and systemic silencing was monitored by the appearance of bleaching in the agroinfiltrated area or near the leaf veins, respectively. Interestingly, all plants agroinfiltrated in the basal region of the leaf displayed SS, although with a slight delay and to a lower intensity compared to plants agroinfiltrated in the whole leaf surface (Figure 3). By contrast, plants agroinfiltrated in the distal region of the leaf did not display SS (Figure 3). These results indicate that amiR‐NbSu‐2 must be expressed in leaf areas proximal to the petiole for triggering SS.

**Figure 4:**
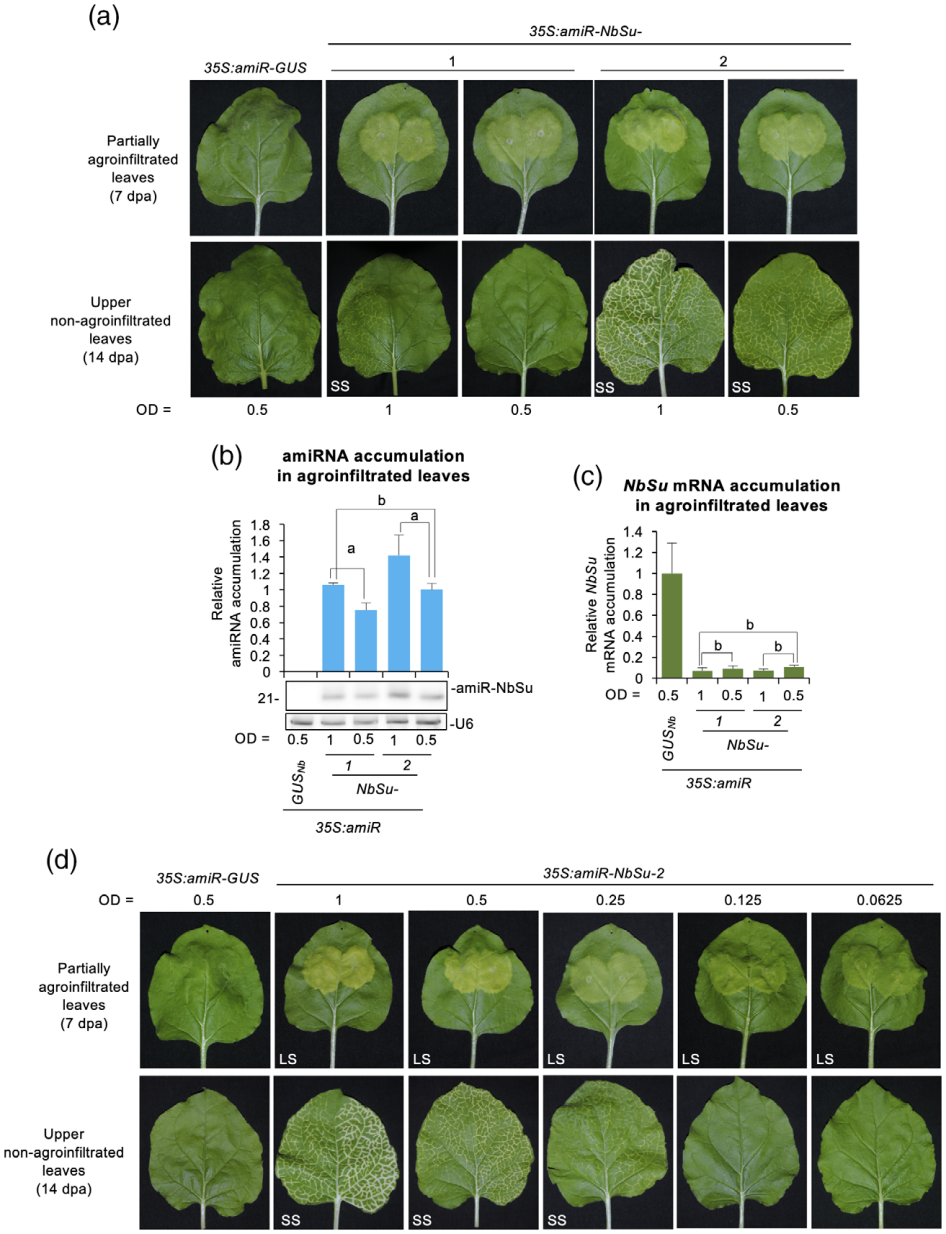
Effects of amiRNA expression level on systemic silencing induction. (a) Photographs of partially agroinfiltrated leaves at 7 days post‐agroinfiltration (dpa) (top) and of upper non‐agroinfiltrated leaves at 14 dpa (bottom). amiRNA constructs were agroinfiltrated to a final optical density (OD) of 1 or 0.5, as indicated. Other details are as shown in Figure 2(a). (b) Northern blot detection of amiR‐NbSu amiRNAs in RNA preparations from agroinfiltrated leaves at 2 dpa. amiRNA constructs were agroinfiltrated to a final OD of 1 or 0.5, as indicated. The graph at top shows the mean ± SD (*n* = 3) amiR‐NbSu relative accumulation (*35S:amiR‐NbSu‐1* = 1.0). Bars with the letter ‘a’ are significantly different from that of sample *35S:amiR‐NbSu‐2* at OD = 0.5, whereas the bar with the letter ‘b’ is not. Other details are as shown in Figure 1(b). (c) Accumulation of *NbSu* mRNA in agroinfiltrated leaves. Mean ± SE relative level (*n* = 3) of *NbSu* mRNAs after normalization to *PROTEIN PHOSPHATASE 2A* (*PP2A*), as determined by quantitative RT‐PCR (qPCR) [*35S:amiR‐GUS*_*Nb*_ (OD = 0.5) = 1.0 in all comparisons]. Other details are as shown in Figure 1(b). (d) Photographs of partially agroinfiltrated leaves at 7 dpa (top) and of upper non‐agroinfiltrated leaves at 14 dpa (bottom). amiRNA constructs were agroinfiltrated to a final OD of 1, 0.5, 0.25, 0.125 or 0.0625, as indicated. Other details are as shown in Figure 2(a). [Colour figure can be viewed at wileyonlinelibrary.com]

Next, we investigated whether the appearance or intensity of systemic silencing induced by amiR‐NbSu‐1 and amiR‐NbSu‐2, respectively, could be correlated with the level of expression of the amiRNA in agroinfiltrated leaves. For that purpose, *35S:amiR‐NbSu‐1* and *35S:amiR‐NbSu‐2* were agroinfiltrated as before at two different final optical densities (ODs): 0.5 (the OD used in previous experiments) and 1.0. *35S:amiR‐GUS*_*Nb*_ was also agroinfiltrated at 0.5 as negative control. In addition, constructs were also independently agroinfiltrated in two areas of two leaves of three plants to better visualize the degree of bleaching as a result of the high color contrast between yellow agroinfiltrated areas and dark green non‐infiltrated areas. At 7 dpa, all agroinfiltrated areas were bleached except those agroinfiltrated with *35S:amiR‐GUS*_*Nb*_ (Figure 4a, top). At 14 dpa, near‐vein bleaching was more intense in upper leaves of plants with leaves fully agroinfiltrated with *35S:amiR‐NbSu‐2* at the highest OD (OD = 1) (Figure 4a, bottom). As before, no SS was observed in upper leaves of plants agroinfiltrated with *35S:amiR‐NbSu‐1* at OD = 0.5. However, mild near‐vein bleaching was detected in upper leaves of plants agroinfiltrated with *35S:amiR‐NbSu‐1* at OD = 1 (Figure 4a, bottom). RNA blot assays of RNA preparations from agroinfiltrated areas showed that, for both amiRNAs, amiRNA accumulation significantly increased with the OD at which the construct was agroinfiltrated (Figure 4b). Remarkably, near‐vein bleaching intensity in upper leaves positively correlated with amiRNA accumulation in agroinfiltrated leaves. For example, the strongest SS was observed in upper leaves of plants accumulating the highest amount of amiRNA in agroinfiltrated leaves, as observed in plants expressing *35S:amiR‐NbSu‐2* at OD = 1. Interestingly, plants expressing *35S:amiR‐NbSu‐1* at OD = 1 and *35S:amiR‐NbSu‐2* at OD = 0.5 accumulated similar (intermediate) amounts of amiRNA in agroinfiltrated tissues (Figure 4b), although upper leaves of amiR‐NbSu‐2 expressing plants displayed more intense bleaching (Figure 4a, bottom). Finally, RT‐qPCR analysis confirmed the significant decrease of *NbSu* mRNA in leaves agroinfiltrated with any of the two anti‐*NbSu* amiRNAs at any of the two ODs (Figure 4c). Still, for both amiRNAs, no significant differences in *NbSu* mRNA accumulation were observed when the amiRNA was expressed to a different OD (Figure 4c).

To further confirm that induction of SS was positively correlated with the level of amiRNA expression, *35S:amiR‐NbSu‐2* was agroinfiltrated at final ODs of 1, 0.5, 0.25, 0.125 and 0.0625. As before, *35S:amiR‐GUS*_*Nb*_ was also agroinfiltrated at OD = 0.5 as negative control. Visual analysis of agroinfiltrated areas revealed that all areas expressing *35S:amiR‐NbSu‐2* showed the bleaching phenotype, although the degree of bleaching was progressively reduced as the amiRNA was agroinfiltrated to a lower OD (Figure 4d). By contrast, systemic near‐vein bleaching was only observed in upper leaves of plants agroinfiltrated in the whole leaf surface with *35S:amiR‐NbSu‐2* to an OD ≥ 0.25 (Figure 4d). Moreover, the intensity and spread of bleaching in upper leaves gradually decreased with the OD at which the construct was agroinfiltrated (Figure 4d). Taken together, these results indicate that the degree of SS positively correlates with the level of expression of the trigger amiRNA in the agroinfiltrated tissue.

### Secondary sRNAs are not the mobile signal

**Figure 5:**
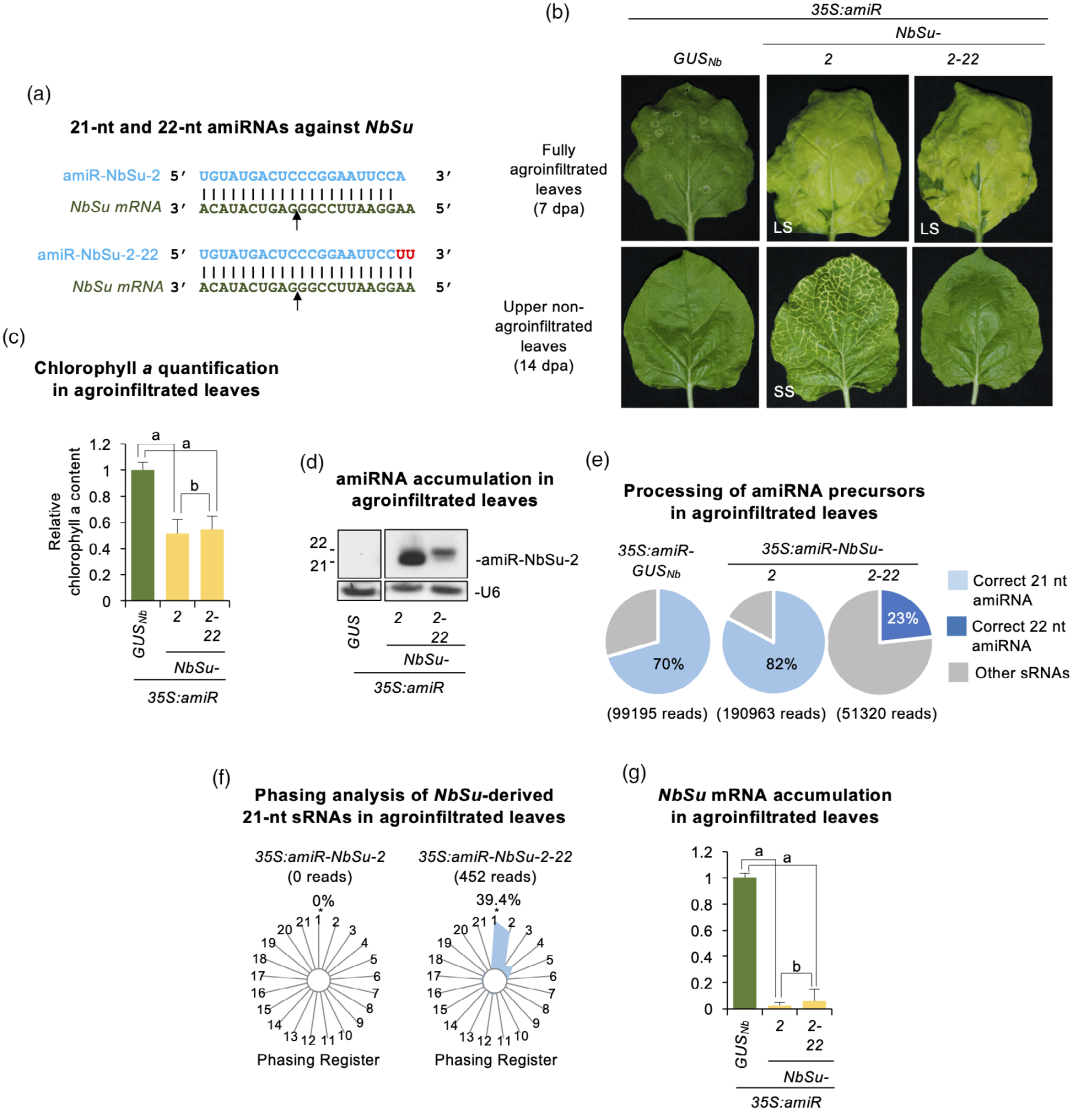
Comparative analysis of silencing effects triggered by 21‐ or 22‐nucleotide forms of amiR‐NbSu‐2. (a) Base‐pairing of ‐amiRNAs and *NbSu* target mRNAs. Nucleotides changed or added respect to amiR‐NbSu‐2 are in red. Other details are as shown in Figure 1(a). (b) Top: photographs at 7 days post‐agroinfiltration (dpa) of leaves agroinfiltrated with the different amiRNA constructs. Bottom: photographs at 14 dpa of upper non‐agroinfiltrated leaves from plants agroinfiltrated with the different constructs. (c) Bar graph showing the relative content of chlorophyll *a* in agroinfiltrated areas (*35S:amiR‐GUS*_*Nb*_ = 1.0). Other details are as shown in Figures 1(c) and 2(a). (d) Northern blot detection of amiR‐NbSu‐2 21‐ and 22‐nucleotide forms in RNA preparations from agroinfiltrated leaves at 2 dpa. (e) amiRNA processing from *AtMIR390a*‐based precursors. Pie charts show percentages of reads corresponding to expected, accurately processed 21‐ or 22‐nucleotide mature amiRNAs (light or dark gray sections, respectively) or to other 19–24‐nucleotide sRNAs (gray sectors). (f) Phasing analysis of *NbSu*‐derived 21‐nucleotide sRNAs. Radar plots show proportions of 21‐nucleotide reads corresponding to each of the 21 registers from *AtTAS1c* transcripts, with position 1 designated as immediately after the amiR‐NbSu‐2/amiR‐NbSu‐2‐22 guided cleavage site. The percentage of 21‐nucleotide reads corresponding to phasing register 1 is indicated. (g) Accumulation of *NbSu* mRNA in agroinfiltrated leaves. Other details are as shown in Figure 1(e). [Colour figure can be viewed at wileyonlinelibrary.com]

In some cases, sRNA‐mediated cleavage of target RNAs can trigger the production of phased 21‐nucleotide secondary sRNAs from DCL4‐processed dsRNAs synthesized by RDR6 complexes from one of the target cleaved products. This process of transitivity expands the set of sRNAs that can regulate a particular target and indeed enhances both cell autonomous and systemic silencing (Felippes and Waterhouse, 2020)]. In this scenario, we hypothesized that both local and systemic silencing of *NbSu* might be explained, at least in part, by the activity of secondary sRNAs produced from *NbSu* mRNAs upon amiR‐NbSu‐2 targeting. Because transitivity is typically triggered by 22‐nucleotide sRNAs (Chen et al., 2010; Cuperus et al., 2010), we reasoned that 22‐nucleotide forms of amiR‐NbSu‐2 could enhance both local and systemic silencing. To this purpose, we engineered the *35S:amiR‐NbSu‐2‐22* construct for expressing a 22‐nucleotide form of amiR‐NbSu‐2 (amiR‐NbSu‐2‐22) from a modified *AtMIR390a* precursor as described previously (Cuperus et al., 2010) (Figure 5a). The amiR‐NbSu‐2‐22 was engineered to fully base pair with *NbSu* mRNA to further enhance transitivity (Figure 5a).

*35S:amiR‐NbSu‐2‐22* was agroinfiltrated in two full leaves of each of three independent plants. For comparative purposes, *35S:amiR‐NbSu‐2* expressing a 21‐nucleotide form of amiR‐NbSu‐2 (Figure 1d) was also tested (Figure 5a), as well as the control construct expressing amiR‐GUS_Nb_. At 7 dpa, all leaves expressing 21‐ or 22‐nucleotide forms of amiR‐NbSu‐2 showed similar bleaching phenotype (Figure 5b) and chlorophyll content (Figure 5c). By contrast, only upper leaves of plants expressing 21‐nucleotide amiR‐NbSu‐2 showed near‐vein bleaching (Figure 5b). RNA blot analysis of RNA preparations from agroinfiltrated leaves showed that amiR‐NbSu‐2‐22 accumulated mainly as 22‐nucleotide sRNA species, and to apparently lower levels than 21‐nucleotide amiR‐NbSu‐2 (Figure 5d). To confirm the correct processing of amiRNA precursors and analyze the presence of *NbSu*‐derived secondary sRNAs, sRNA libraries from leaves expressing *35S:amiR‐GUS*_*Nb*_, *35S:amiR‐NbSu‐2* and *35S:amiR‐NbSu‐2‐22* were prepared and sequenced. In samples expressing *35S:amiR‐NbSu‐2*, the majority (82%) of 19–24‐nucleotide (+) reads corresponded to authentic 21‐nucleotide amiR‐NbSu‐2 (Figure 5e), whereas only 0.001% of the reads corresponded to misprocessed 22‐nucleotide forms. By contrast, only 23% of the reads from samples expressing *35S:amiR‐NbSu‐2‐22* corresponded to correct 22‐nucleotide forms, confirming our ability to produce authentic 22‐nucleotide forms of amiR‐NbSu‐2 *in vivo*. Unexpectedly, 57% of reads in *35S:amiR‐NbSu‐2‐22* samples corresponded to 21‐nucleotide forms of amiR‐NbSu‐2, which were not observed in the northern blot assay (Figure 5d). Still, it cannot be ruled out that sRNA library preparation and/or sequencing protocols contain biases that may preferentially increase the final number of 21‐nucleotide reads corresponding to amiR‐NbSu‐2. In any case, it should be noted that the lower proportion of correct 22‐nucleotide forms compared to 21‐nucleotide forms of amiR‐NbSu‐2 from *AtMIR390a*‐based precursors was expected because similar results were obtained when expressing 21‐nucleotide and 22‐nucleotide forms of *A. thaliana* miR173 (Cuperus et al., 2010). Finally, in *35S:amiR‐GUS*_*Nb*_‐expressing leaves, the majority (70%) of the reads corresponded to authentic 21‐nucleotide amiR‐GUS_Nb_ (Figure 5e) supporting the correct processing of *AtMIR390a‐GUS*_*Nb*_ precursors.

Next, we analyzed the presence of predicted *NbSu*‐derived secondary sRNAs in phase with amiR‐NbSu‐2 or amiR‐NbSu‐2‐22 cleavage sites in agroinfiltrated leaves expressing *35S:amiR‐NbSu‐2* or *35S:amiR‐NbSu‐2‐22*, respectively (Figure 5f, Data S1). No reads corresponding to 21‐nucleotide predicted phased sRNAs were observed among the sRNA reads sequenced from *35S:amiR‐NbSu‐2*‐expressing leaves and mapping to *NbSu* (Data S1). By contrast, 39.4% of 21‐nucleotide sRNA reads from leaves expressing *35S:amiR‐NbSu‐2‐22* were in register with amiR‐NbSu‐2‐22 cleavage site (phasing register of 1) (Figure 5f, Data S1), confirming the presence of *NbSu*‐derived secondary sRNAs only in *35S:amiR‐NbSu‐2‐22* samples. Taken together, these results indicate that the accumulation of *NbSu*‐derived secondary siRNAs triggered by amiR‐NbSu‐2‐22 does not induce SS. Finally, *NbSu* target mRNA accumulation analyzed by RT‐qPCR was drastically reduced in agroinfiltrated leaves expressing either 21‐ or 22‐nucleotide forms of amiR‐NbSu‐2 (Figure 5g).

To further confirm that the silencing effects induced by amiR‐NbSu‐2 were not caused by secondary sRNAs produced from targeted *NbSu* mRNAs, *35S:amiR‐NbSu‐2* and *35S:amiR‐GUS*_*Nb*_ were agroinfiltrated in the whole surface of two leaves of three independent *N. benthamiana* DCL4i and RDR6i knockdown plants as well as in wild‐type plants (Figure S1). DCL4i and RDR6i plants accumulate low levels of DCL4 and RDR6 respectively (Dadami et al., 2013; Schwach et al., 2005) (Figure S1a), which are key components of secondary sRNA biogenesis pathways. As before, constructs were also infiltrated in two areas of two leaves of three independent plants for each plant genotype for better visualization of the bleaching phenotype. Similar bleached phenotypes were observed both in agroinfiltrated areas and upper non‐agroinfiltrated leaves of wild‐type, DCL4i or RDR6i plants expressing *35S:amiR‐NbSu‐2*, whereas no bleached phenotypes were observed in control agroinfiltrations (Figure S1b). Therefore, these results strongly suggest that neither the local nor the systemic silencing of *NbSu* is caused by *NbSu*‐derived secondary sRNAs.

### amiR‐NbSu‐2 moves from agroinfiltrated tissue to upper, distal silenced tissues

**Figure 6:**
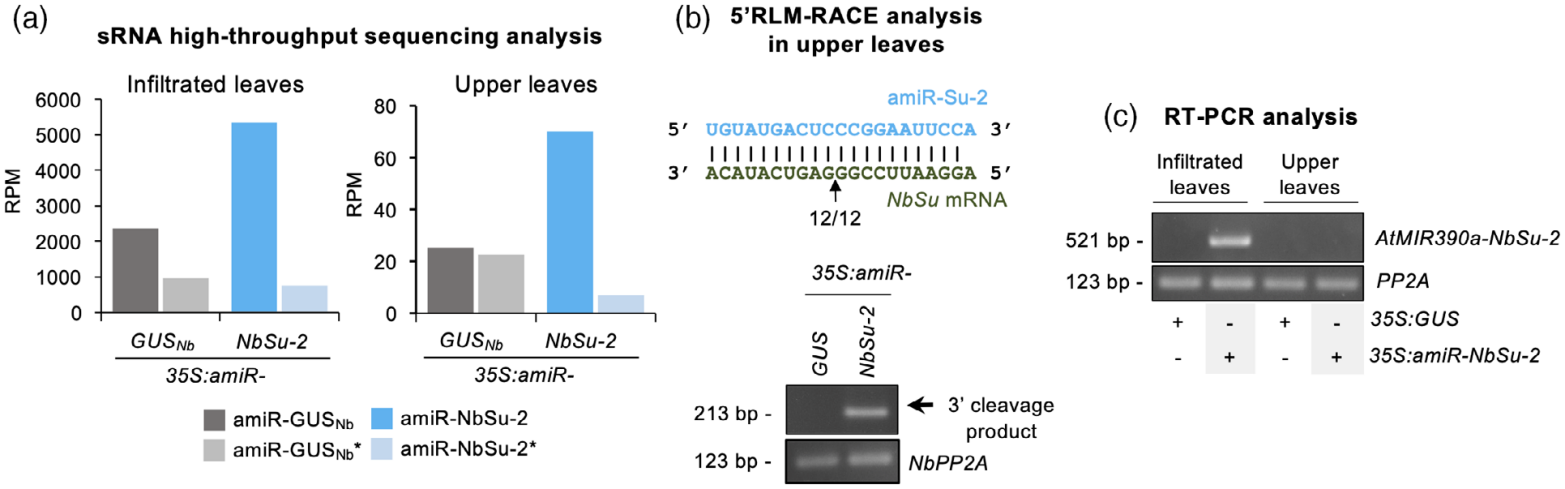
Analysis of amiRNA presence and activity in distal tissues. (a) Bar graph showing the accumulation (reads per million, RPM) of both amiRNA duplex strands revealed by high‐throughput sequencing of small RNA libraries prepared from agroinfiltrated leaves (left) or from upper non‐agroinfiltrated leaves (right). Bars representing amiR‐GUS_Nb_ guide and star strands RPMs are in dark and light gray, respectively. Bars representing amiR‐NbSu‐2 guide and star strands RPMs are in dark and light blue, respectively. (b) RT‐PCR detection of *AtMIR390a‐NbSu‐2* precursors in agroinfiltrated or upper non‐agroinfiltrated leaves (top). RT‐PCR products corresponding to the control *NbPP2A* are also shown (bottom). (c) 5′‐RLM‐RACE analysis of amiR‐NbSu‐2‐guided cleavage of *NbSu* in upper leaves. Top: the predicted base‐pairing between amiR‐NbSu‐2 and *NbSu* mRNA is shown, and the expected amiR‐NbSu‐2‐based cleavage site is indicated by an arrow. The proportion of cloned 5′‐RLM‐RACE products at the at the expected cleavage site is shown for amiR‐NbSu‐2‐expressing leaves. Bottom: ethidium bromide‐stained gel shows 5′‐RLM‐RACE products corresponding to the 3′ cleavage product from amiR‐NbSu‐2‐guided cleavage (top gel) and RT‐PCR products corresponding to the control *NbPP2A* gene (bottom gel). The position and size of the expected amiRNA‐based 5′‐RLM‐RACE products are indicated, as well as the position and size of control RT‐PCR products. [Colour figure can be viewed at wileyonlinelibrary.com]

Next, we reasoned that the SS of *NbSu* in *35S:amiR‐NbSu‐2*‐expressing plants could be the direct result of amiR‐NbSu‐2 cleavage activity in upper tissues. First, to check the presence of amiR‐NbSu‐2 in upper bleached tissues, sRNA libraries were prepared from leaf areas including the near‐vein bleached phenotype, as well as control libraries from upper tissues of amiR‐GUS_Nb_‐expressing plants. Reads corresponding to amiR‐NbSu‐2 were detected to a relatively high number [70 reads per million (RPM)] in upper leaves, although this value was clearly lower than that of reads from agroinfiltrated leaves (5338 RPM) (Figure 6a). Importantly, amiR‐NbSu‐2 reads were essentially absent in both agroinfiltrated and upper leaves from plants expressing amiR‐GUS_Nb_ (Figure S2), thus confirming the specificity of the sequencing results. The presence of amiR‐GUS_Nb_ was also confirmed both in agroinfiltrated leaves and upper leaves from plants expressing amiR‐GUS_Nb_ but not from plants expressing amiR‐NbSu‐2 (Figure S2). Interestingly, reads corresponding to amiRNA star strands (amiRNA*) of both amiRNAs were detected in agroinfiltrated and in upper leaves (Figure 6a). Thus, these results indicate that amiRNA duplexes are present in upper tissues. Furthermore, to check amiR‐NbSu‐2 cleavage activity in distal tissues, 5′‐RLM‐RACE analysis was performed in upper leaves of amiR‐NbSu‐2‐expressing plants showing the near‐vein bleaching phenotype (Figure 6b). For control purposes, the same analysis was carried out in similar leaves of plants agroinfiltrated with *35S:amiR‐GUS*_*Nb*_. 3′ cleavage products of the expected size (213 bp) were detected in samples expressing amiR‐NbSu‐2‐ but not in samples from amiR‐GUS_Nb_‐expressing plants (Figure 6b). Sequencing analysis confirmed that all (12/12) the sequences comprising these products contained a canonical 5′ end position predicted for amiR‐NbSu‐2‐guided cleavage (Figure 6b). These results indicate that amiR‐NbSu‐2 is cleaving *NbSu* mRNAs at the predicted position in upper non‐agroinfiltrated tissues.

Finally, we reasoned that the presence of amiR‐NbSu‐2 in upper tissues could result from the movement of the amiRNA precursor or of the amiRNA duplex itself from the agroinfiltrated leaves. To clarify the identity of the mobile molecule(s) causing SS, the presence of 521‐nucleotide *AtMIR390a‐NbSu‐2* amiRNA precursors was analyzed by RT‐PCR in both source and recipient tissues. *AtMIR390a‐NbSu‐2* precursors were detected in RNA preparations of infiltrated leaves but not from those from upper leaves (Figure 6c), thus suggesting that amiR‐NbSu‐2 duplexes and not their precursors may be the mobile entities causing SS.

### SS can also be triggered by a 21‐nucleotide syn‐tasiRNA

**Figure 7:**
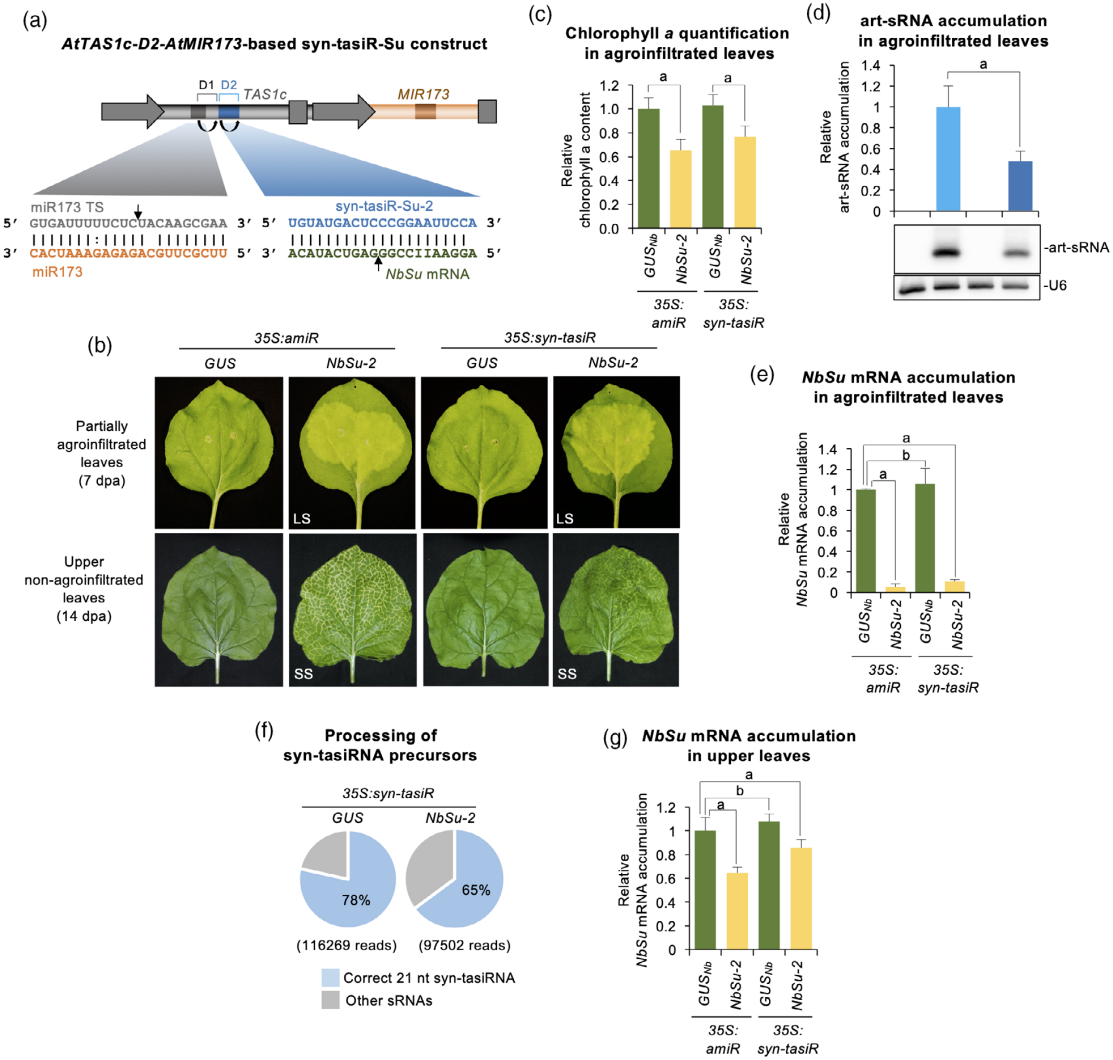
Functional analysis of synthetic *trans*‐acting small interfering RNAs (syn‐tasiRNAs) against *N. benthamiana SULFUR* (amiR‐NbSu) in agroinfiltrated and upper leaves. (a) Diagram of the *35S:syn‐tasiR‐Su‐2* construct. *AtMIR173* and miR173 sequences are shown in light and dark brown, respectively, whereas syn‐tasiR‐NbSu‐2 and NbSu mRNA sequences are in blue and green, respectively. tasiRNA positions 3′D1[+] and 3′D2[+] are indicated by brackets, with position 3′D2[+] highlighted in blue. Curved black arrows indicate DCL4 processing sites. Black linear arrow indicate sRNA‐guided cleavage sites. ts refers to target site. (b) Photographs of partially agroinfiltrated leaves at 7 days post‐agroinfiltration (dpa) (top) and of upper non‐agroinfiltrated leaves at 14 dpa (bottom). Other details are as shown in Figure 2(a). (c) Bar graph showing the relative content of chlorophyll *a* in agroinfiltrated areas (*35S:amiR‐GUS*_*Nb*_ = 1.0). Other details are as shown in Figures 1(c) and 2(a). (d) Northern blot detection of artificial small RNAs (art‐sRNAs) against *NbSu* in RNA preparations from agroinfiltrated leaves at 2 dpa. The graph at top shows the mean ± SD (*n* = 3) art‐sRNA relative accumulation (*35S:amiR‐NbSu‐2* = 1.0). Bar with the letter ‘a’ is significantly different from that of sample *35S:amiR‐NbSu‐2*. Other details are as shown in Figure 1(c). (e) Accumulation of *NbSu* mRNA in agroinfiltrated leaves. Other details are as shown in Figure 1(e). (f) Syn‐tasiRNA processing from *AtTAS1c*‐based precursors. Pie charts show percentages of reads, with the percentage of 21‐nucleotide reads of accurately processed mature syn‐tasiRNAs indicated in the blue sectors. Gray sectors represent the percentage of 19–24‐nucleotide reads of other small RNAs. (g) Accumulation of *NbSu* mRNA in upper non‐agroinfiltrated leaves. Other details are as shown in Figure 1(e). [Colour figure can be viewed at wileyonlinelibrary.com]

Mobility of plant miRNAs and tasiRNAs may differ according to previous reports (de Felippes et al., 2011), and tasiRNAs have only been shown to move short‐range as non‐cell‐autonomous signals (Chitwood et al., 2009). Here, we investgated whether syn‐tasiR‐NbSu‐2, a syn‐tasiRNA of identical sequence to amiR‐NbSu‐2, was able to trigger SS. To that purpose, the *35S:syn‐tasiR‐NbSu‐2* construct was generated, as well as the *35S:syn‐tasiR‐GUS*_*Nb*_ control construct aimed to express syn‐tasiR‐GUS_Nb_, a syn‐tasiRNA with identical sequence to that of amiR‐GUS_Nb_ (Figure 7a). Both constructs were independently agroinfiltrated in *N. benthamiana* plants as explained before, and in parallel with *35S:amiR‐NbSu‐2* for control purposes. At 7 dpa, areas agroinfiltrated with *35S:amiR‐NbSu‐2* or *35S:syn‐tasiR‐NbSu‐2* showed comparable bleaching degree (Figure 7b), which was confirmed by a similar decrease in chlorophyll levels in these areas (Figure 7c). Interestingly, amiR‐NbSu‐2 accumulated to significantly higher levels than syn‐tasiR‐NbSu‐2 (Figure 7d) but induced comparable downregulation of *NbSu* mRNA in agroinfiltrated leaves (Figure 7e). To confirm the correct processing of *AtTAS1c*‐based syn‐tasiRNA precursors, sRNA libraries from leaves expressing *35S:syn‐tasiR‐GUS*_*Nb*_ and *35S:syn‐tasiR‐NbSu‐2* were prepared and sequenced. Both precursors were processed accurately, because 78 and 65% of the reads corresponded to authentic 21‐nucleotide syn‐tasiR‐GUS_Nb_ and syn‐tasiR‐NbSu‐2, respectively (Figure 7f). Importantly, systemic near‐vein bleaching was observed in upper leaves from plants expressing *35S:syn‐tasiR‐NbSu‐2*, although with a lower intensity than in similar leaves from *35S:amiR‐NbSu‐2*‐expressing plants (Figure 7b). Not unexpectedly, *NbSu* mRNA accumulation was significantly lower in distal bleached tissues from plants expressing amiR‐NbSu‐2 compared to plants expressing syn‐tasiR‐NbSu‐2 (Figure 7g). Also, as shown for amiR‐NbSu‐2, syn‐tasiR‐NbSu‐2 must also be expressed in leaf areas proximal to the petiole for triggering SS (Figure S3). Finally, we tested the speed of syn‐tasiR‐NbSu‐2 and amiR‐NbSu‐2 movement by removing the agroinfiltrated leaves 1, 2, 3, 4 or 5 dpa and scoring the say of near‐vein leaf chlorosis appearance in apical leaves. For syn‐tasiR‐NbSu‐2, 50 and 100% of plants exhibited SS when the agroinfiltrated leaves were removed 2 and 3 dpa, respectively, whereas all plants in which the *35S:amiR‐NbSu‐2*‐expressing leaves were removed at 2 dpa showed SS (Figure S4). These results suggest that the production and translocation of the amiRNA and syn‐tasiRNA duplexes occurs within 1–2 and 1–3 dpa, respectively.

**Figure 8:**
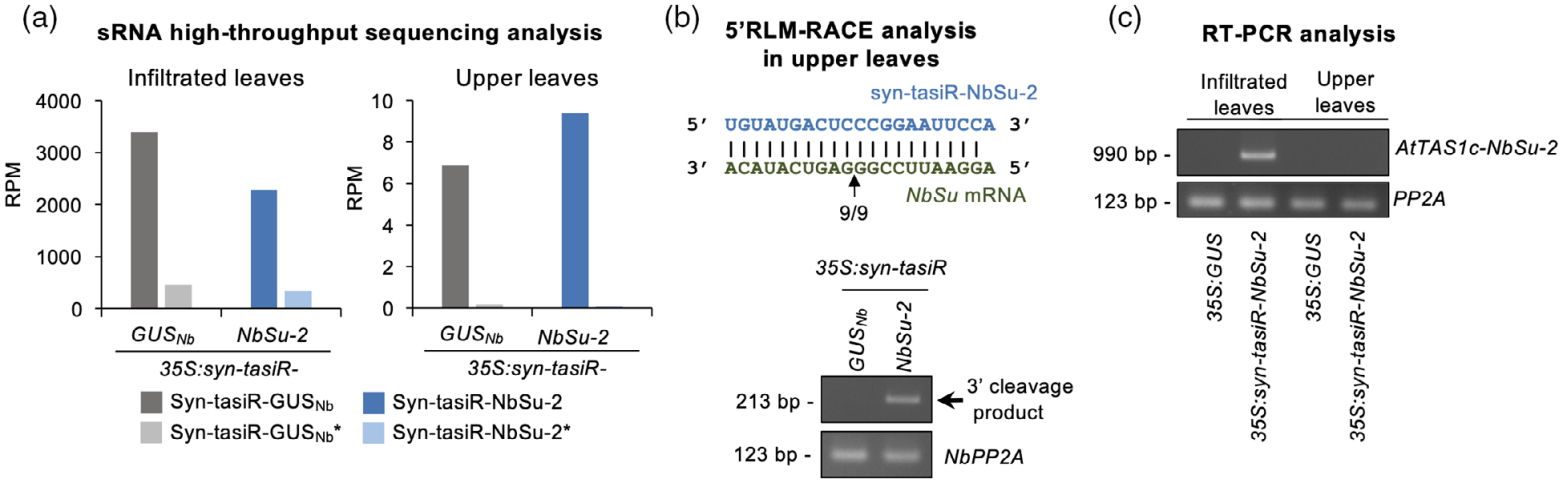
Analysis of syn‐tasiRNA presence and activity in distal tissues. (a) Bar graph showing the accumulation (reads per million, RPM) of both syn‐tasiRNA duplex strands revealed by high‐throughput sequencing of small RNA libraries prepared from agroinfiltrated leaves (left) or from upper non‐agroinfiltrated leaves (right). Bars representing syn‐tasiR‐GUS_Nb_ guide and star strands RPMs are in dark and light gray, respectively. Bars representing syn‐tasiR‐NbSu‐2 guide and star strands RPMs are in dark and light blue, respectively. (b) 5′‐RLM‐RACE analysis of syn‐tasiR‐NbSu‐2‐guided cleavage of *NbSu* in upper leaves. Top: the predicted base‐pairing between syn‐tasiR‐NbSu‐2 and *NbSu* mRNA is shown, and the expected syn‐tasiR‐NbSu‐2‐based cleavage site is indicated by an arrow. Other details are as shown in Figure 5(c). (c) RT‐PCR detection of *AtTAS1c‐NbSu‐2* precursors in agroinfiltrated or upper non‐agroinfiltrated leaves (top). Other details are as shown in Figure 5(b). [Colour figure can be viewed at wileyonlinelibrary.com]

To analyze the presence of syn‐tasiRNAs in upper tissues, sRNA libraries were prepared from near‐vein bleached samples of syn‐tasiR‐NbSu‐2‐expressing plants, together with control libraries from similar tissues of syn‐tasiR‐GUS_Nb_‐expressing plants. Reads corresponding to syn‐tasiR‐NbSu‐2 were detected in upper leaves (9.4 RPM), although to a much lower number than in agroinfiltrated leaves (2280 RPM) (Figure 8a), and were essentially absent in *35S:syn‐tasiR‐GUS*_*Nb*_ samples (Figure S5). The presence syn‐tasiR‐GUS_Nb_ was also confirmed in upper (6.9 RPM) and in agroinfiltrated tissues (3399 RPM), and was negligible in *35S:syn‐tasiR‐NbSu‐2* samples (Figure S5). Reads corresponding to syn‐tasiRNA star strands (syn‐tasiRNA*) of both syn‐tasiRNA species were also detected in both agroinfiltrated and upper tissue, thus indicating the syn‐tasiRNA duplexes are present in upper tissues. 5′‐RLM‐RACE analysis confirmed the presence of 3′ products derived from syn‐tasiR‐NbSu‐2 cleavage of *NbSu* mRNAs in near‐vein bleached tissues (Figure 8b). Finally, *AtTAS1c‐NbSu‐2* precursors could only be detected in agroinfiltrated tissues but not in upper leaves (Figure 8c). All together, these results suggest that 21‐nucleotide syn‐tasiRNA duplexes can also move from transiently expressing tissues to upper distal parts of the plant to silence target mRNAs.

## DISCUSSION

Here, we report the ability of two classes of 21‐nucleotide art‐sRNAs, such as amiRNAs and syn‐tasiRNAs, to move throughout the plant away from their production sites and systemically silence a plant endogenous gene. Both amiR‐NbSu‐2 and syn‐tasiR‐NbSu‐2 were agroinfiltrated in the whole surface of *N. benthamiana* leaves and induced the SS of *NbSu*, which was easily detected as a strong chlorosis near the veins of upper distal leaves. Importantly, we were able to visualize the SS of *NbSu* because of the characteristic bleaching phenotype derived from *NbSu* downregulation, which is clearly visible to the naked eye. In this sense, the *N. benthamiana*/amiR‐NbSu‐2 and *N. benthamiana*/syn‐tasiR‐NbSu‐2 experimental systems may represent valuable tools with a clear and visible readout for the analysis of determinants controlling the induction and/or spread of the SS triggered by plant sRNAs.

Our initial experiments comparing the induction of local and systemic silencing by amiR‐NbSu‐1 and amiR‐NbSu‐2 showed that, although local silencing efficiency was similar for both amiRNAs, SS was triggered only by amiR‐NbSu‐2. Because amiR‐NSu‐2 accumulated to significantly higher levels compared to amiR‐NbSu‐1 in agroinfiltrated tissues, we investigated whether the degree of SS was dependent on the local accumulation level of the amiRNA. Subsequent experiments aiming to test SS induction upon agroinfiltration of amiRNA constructs to different ODs showed that the intensity of SS triggered by amiR‐NbSu‐2 was indeed positively correlated with the expression level of amiR‐NbSu‐2 in agroinfiltrated leaves. Moreover, amiR‐NbSu‐1 was able to trigger SS only when expressed at the higher OD tested (OD = 1), thus highlighting that the amiRNA expression level is a critical factor for SS induction. Similarly, the systemic movement of siRNAs has also been positively correlated with their expression level, such that a higher copy number of a triggering transgene led to more efficient acquired silencing (Palauqui & Balzergue, 1999).

Another critical factor controlling SS induction could be the place of expression of the art‐sRNA. Previously, the limited entry of endogenous miRNAs into the phloem suggested that the expression in phloem companion cells might be a requirement for long‐distance transport of miRNAs (Skopelitis et al., 2018; Subramanian, 2019). Indeed, long‐distance signaling miRNAs such as miR395 and miR399, known to control shoot‐to‐root communication upon S and P starvation, respectively, are expressed in companion cells of the phloem (Kawashima et al., 2009) and in several vascular tissues including the phloem (Aung et al., 2006). In our experiments, amiR‐NbSu‐2 and syn‐tasiR‐NbSu‐2 triggered SS only when agroinfiltrated in areas neighboring the leaf petiole but not in distal regions of the leaf. Thus, it is likely that amiR‐NbSu‐2 and syn‐tasiR‐NbSu‐2, when expressed near the petiole, can reach the companion cells and be loaded into the sieve elements of the phloem for long‐distance movement. It should be noted that, although the movement of a SS signal can be bidirectional in plants (Voinnet et al., 1998), under our experimental conditions, we could only detect near‐vein chlorosis in leaves above those agroinfiltrated with *35S:amiR‐NbSu‐2* or *35S:syn‐tasiR‐NbSu‐2*, but not in lower leaves (Table S1). This is probably a reflection of the pattern of spread of art‐sRNA duplexes following the movement of photoassimilates or viruses in plants, from source (older) to sink (younger) leaves (Leisner & Turgeon, 1993; Voinnet et al., 1998).

Although a few miRNAs can move systemically in plants (see below), siRNAs are considered to be more mobile at long distances. For example, secondary siRNAs generated from transgenes or from invading viruses propagate the spread of silencing from a single leaf systemically throughout the plant, and siRNAs (especially those of 24 nucleotides) travel systemically to direct DNA methylation of transposable elements in target tissues, including meristematic and meiotically active cells (Liu & Chen, 2018; Molnar et al., 2010). However, tasiRNA mobility appears to be more limited, as exemplified by miR390a‐*TAS3*‐dependent tasiRNA‐AUXIN RESPONSE FACTORs (i.e. tasi‐ARFs) that are produced in the upper side of the leaf and diffuse to create a gradient that patterns the adaxial–abaxial axis of leaves (Chitwood et al., 2009). Thus, our results showing syn‐tasiRNA systemic movement indicate that tasiRNAs can actually move long‐distances, and it is possible that the overexpression of syn‐tasiR‐NbSu‐2 in our experiments has facilitated this.

An interesting result is that amiR‐NbSu‐2 and syn‐tasiR‐NbSu‐2, despite having identical sequences, caused distinct degrees of SS. SS triggered by amiR‐NbSu‐2 was more intense and extended much further than that induced by syn‐tasiR‐NbSu‐2. Which factor(s) could explain the differences in SS intensity caused by both classes of art‐sRNAs? The simplest explanation may be that the accumulation level of the art‐sRNA plays a critical role in this matter because amiR‐NbSu‐2 accumulated in agroinfiltrated source tissues to significantly higher levels compared to syn‐tasiR‐NbSu‐2, which may facilitate its earlier entry to the phloem stream (Figure S4). Still, we cannot rule out the possibility that other factors such as the specific genetic requirements for amiRNA and syn‐tasiRNA biogenesis contribute to these differences. Interestingly, Arabidopsis transgenic plants expressing a syn‐tasiRNA against *SULFUR* (tasiR‐SUL) from the companion cell‐expressing *SUC2* promoter have extended bleaching compared to plants expressing an amiRNA (amiR‐SUL) of identical sequence from the same promoter, despite tasiR‐SUL accumulating to significantly lower levels compared to amiR‐SUL (de Felippes et al., 2011). In this case, more secondary sRNAs were detected in tasiR‐SUL plants than in amiR‐SUL plants, which was proposed to contribute to the spreading of tasiR‐SUL triggered silencing (de Felippes et al., 2011). Because tasiRNA biogenesis occurs on membrane‐bound polysomes (Hou et al., 2016; Li et al., 2016), it has also been suggested that this may have facilitated tasiR‐SUL delivery to adjacent cells through plasmodesmata, which are extensions of cellular membranes (Liu et al., 2020). In our experiments, the lack of secondary sRNAs in leaves expressing amiR‐NbSu‐2 or syn‐tasiR‐NbSu‐2 and the transient expression of both art‐sRNA classes may explain the different effects obtained in our system.

It has been proposed that the ability to trigger transitivity (secondary siRNA biogenesis) to amplify and spread the silencing signal to nearby cells may be also a critical factor for effective long‐distance movement of miRNAs (Skopelitis et al., 2018). Indeed, both systemically mobile miR395 and miR399 can trigger transitive silencing (Manavella et al., 2012), as can the majority of the miRNAs that were shown to move from a parasitic plant to a host plant and silence host genes (Shahid et al., 2018). Also, it was shown that transgenic Arabidopsis expressing a 22‐nucleotide but not a 21‐nucleotide amiRNA against *CHALCONE SYNTHASE* (*CHS*) induced widespread silencing of CHS, most likely as a result of the greater mobility of secondary sRNAs and/or the additive effect of both amiRNA and phasiRNA‐directed target mRNA cleavage (McHale et al., 2013). Here, essentially no reads corresponding to 21‐nucleotide predicted phased sRNAs were observed among the sRNA reads mapping to *NbSu* and sequenced from agroinfiltrated and systemically silenced tissues of plants expressing *35S:amiR‐NbSu‐2* or *35S:syn‐tasiR‐NbSu‐2* (Data S1). This indicates that 21‐nucleotide forms of amiR‐NbSu‐2 and syn‐tasiR‐NbSu‐2 do not trigger the production of phased 21‐nucleotide secondary sRNAs neither in agroinfiltrated, nor in upper tissues. By contrast, 22‐nucleotide forms of amiR‐NbSu‐2 triggered transitivity in agroinfiltrated tissues but not SS in distal tissues. Thus, in our system, the ability to trigger transitivity is not a critical factor promoting SS. Indeed, amiR‐NbSu‐2‐22 forms and *NbSu*‐derived phased secondary sRNAs accumulated to much lower levels compared to 21‐nucleotide amiR‐NbSu‐2 forms, which may have limited the capacity of inducing SS. In any case, it is possible that SS induced by 22‐nucleotide amiRNAs might allow target silencing in distal tissues beyond the 10–15 cells but has the drawback of a high risk of off‐target effects because the large population of generated secondary sRNAs may end up targeting other cellular mRNAs besides the intended target RNA (Senthil‐Kumar & Mysore, 2011).

**Figure 9:**
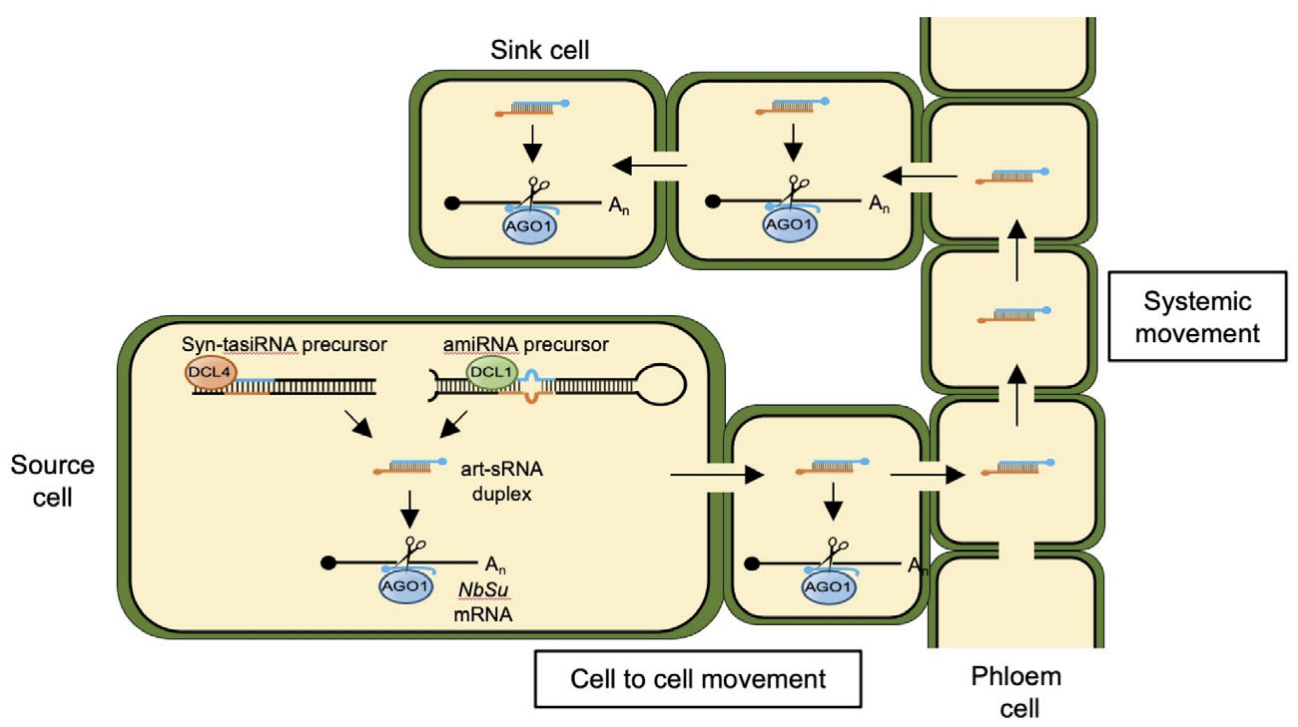
Model describing artificial small RNA (art‐sRNA) biogenesis, silencing activity and movement from source to distal plant tissues. The art‐sRNA duplexes are produced in source cells by DCL1‐ or DCL4‐mediated processing of artificial microRNA (amiRNA) or synthetic *trans*‐acting small interfering RNA (syn‐tasiRNA) precursors, respectively. The guide strand (in blue) of the art‐sRNA duplex is incorporated into ARGONAUTE1 (AGO1) to bind and cleave *NbSu* target mRNAs. The art‐sRNA duplexes most likely move cell to cell through plasmodesmata and long‐distance through the phloem stream using the sieves elements. The art‐sRNA duplexes may exit the sieve elements and move 10–15 cells away. [Colour figure can be viewed at wileyonlinelibrary.com]

Another interesting result is that both amiRNA and syn‐tasiRNA duplexes, and not their precursors, appear to be the molecules moving systemically. Our results seem to support the current prevailing view for both plant miRNAs (Brioudes et al., 2021; Buhtz et al., 2010; Liu & Chen, 2018; Skopelitis et al., 2018) and siRNAs (Devers et al., 2020) that processed sRNAs and not their precursors are the moving entities. Nonetheless, some miRNA precursors such as *MIR156* and *MIR2111* are expressed specifically within the phloem (Tsikou et al., 2018; Yang et al., 2013), and recent results show that (i) miR390b precursors enable the phloem transport of foreign RNA systemically in *N. benthamiana* and (ii) sequences of multiple miRNA precursors are identified in a *Cucurbita maxima* phloem transcriptome (Lezzhov et al., 2019). These observations suggest that, at least for some miRNAs, miRNA phloem signaling might involve the precursor molecules. Moreover, it could be argued that anti‐*NbSu* art‐sRNAs could also be moving systemically bound to AGOs. However, this is unlikely because (i) star strands of both art‐sRNA classes are present in distal tissues; (ii) AGO proteins are systematically absent from Arabidopsis phloem sap proteomes (Batailler et al., 2012; Carella et al., 2016; Guelette et al., 2012); and (iii) AGO proteins generally act cell autonomously and are retained in traversed cells (Devers et al., 2020). In any case, the question of the molecular forms under which plant sRNAs move between cells and long distance is still a matter of study and debate. Here, we propose a model (Figure 9) in which art‐sRNA duplexes are produced in source cells by DCL‐mediated processing of their precursors, next move from cell to cell through plasmodesmata and long‐distance through the phloem stream, and then exit the phloem and move 10–15 cells away to silence target RNAs.

Importantly, the ability of art‐sRNA duplexes of moving from the production site to other distal tissues could be exploited to induce the intentional SS of plant genes and also of pathogenic target RNAs. For example, we recently described a high‐throughput methodology in *N. benthamiana* to identify art‐sRNAs with high antiviral activity (Carbonell & Daròs, 2019), and reported that several amiRNAs and syn‐tasiRNAs against *Tomato spotted wilt virus* (TSWV), expressed in the whole surface of *N. benthamiana* leaves that were further inoculated with TSWV 2 days later, fully protected plants (Carbonell et al., 2019). The complete absence of virus in upper leaves was somewhat unexpected because art‐sRNAs were transiently (and not constitutively) expressed. Considering the results reported in the present study, we now suspect that anti‐TSWV art‐sRNAs were probably moving systemically to the upper leaves and accumulating in a 10–15 cell layer near the veins to block virus exit from the phloem and ultimately prevent its spread. Moreover, this may be the basis of a previously proposed antiviral mechanism in plants in which, upon viral infection, virus derived siRNAs would move from production sites into the phloem to finally reach the meristems and surrounding cells of the shoot apex ahead of the systemically mobile virus (Ratcliff et al., 1997; Schwach et al., 2005). As a consequence, the virus will be suppressed as it enters the meristematic cells and so infection is never established and the plant initiates its recovery (Melnyk et al., 2011). Besides its antiviral function, other observations have highlighted the role of SS as a natural signaling mechanism involved in plant development and physiology. For example, the control of phosphate, copper and sulphate homeostasis relies on the systemic movement of miR399, miR398 and miR395, respectively (Buhtz et al., 2010; Fujii et al., 2005; Matthewman et al., 2012; Pant et al., 2008), whereas the dynamic and systemic fine‐tuning of infection and nodulation by nitrogen and symbiotic rhizobia is controlled by mobile miR2111 (Gautrat et al., 2020; Tsikou et al., 2018).

To conclude, the possibility of expressing art‐sRNAs in a plant tissue and triggering the specific silencing of endogenous plant genes or even pathogenic RNAs in distal tissues in a transitivity‐independent manner has undoubtedly high biotechnological potential, especially when art‐sRNAs are applied exogenously in a GMO‐free way. Indeed, antiviral systemic effects of sprayed dsRNAs in plant leaves have recently be reported (Koch et al., 2016; Mitter et al., 2017). In this context, the exogenous application of amiRNAs or syn‐tasiRNAs would have the inherent advantage of their higher specificity because they function in a transitivity‐independent manner and lead to reduced off‐target risks compared to dsRNA‐based approaches. The optimization of methods for producing and topically applying art‐sRNA precursors is also necessary with respect to the biotechnological use of art‐sRNAs for highly specific silencing at the whole‐individual level in next‐generation crops.

## EXPERIMENTAL PROCEDURES

### Plant materials and growing conditions

*Nicotiana benthamiana* plants were grown in a growth chamber at 25°C under a 12:12 h light/dark photocycle. DCL4i and RDR6i seeds were reported previously (Dadami et al., 2013; Schwach et al., 2005).

### DNA constructs

The amiRNA constructs *35S:amiR‐GUS*_*Nb*_, *35S:amiR‐NbSu‐1*, *35S:amiR‐NbSu‐2*, *35S:amiR‐NbSu‐2‐22* were obtained by ligating annealed oligo pairs D2057/D2058, D2065/D2066, D2067/D2068 and AC‐310/AC‐311, respectively, into *pMDC32B‐AtMIR390a‐B/c* (plasmid 51776; Addgene, Watertown, MA, USA) as described previously (Carbonell et al., 2014). Syn‐tasiRNA construct *35S:syn‐tasiR‐AtTAS1c‐NbSu‐2* was obtained by ligating annealed oligo pair AC‐257/AC‐258 into *pMDC32B‐AtTAS1c‐D2‐B/c‐AtMIR173* (plasmid 137885; Addgene) as described previously (López‐Dolz et al., 2020). *35S:GUS* and *35S:syn‐tasiR‐GUS*_*Nb*_ were reported previously (López‐Dolz et al., 2020; Montgomery, Yoo, et al., 2008). All DNA oligonucleotides used for generating the constructs described above are listed in Table S2.

### amiRNA designs

p‐sams script (https://github.com/carringtonlab/p‐sams) returning unlimited optimal results was used to obtain the complete list of optimal amiRNAs targeting *NbSu* or *E. coli GUS* with high specificity (Data S2). The off‐targeting filtering in *N. benthamiana* transcriptome v5.1 (Nakasugi et al., 2014) was enabled in all amiRNA designs to avoid undesired off‐target effects and to increase specificity.

#### Transient expression of constructs

Agroinfiltration of constructs in *N. benthamiana* leaves was performed as described previously (Cuperus et al., 2010; Llave et al., 2002) using *Agrobacterium tumefaciens* GV3101 strain.

#### RNA preparation

Total RNA from *N. benthamiana* leaves was isolated in extraction buffer (1 m guanidinium thiocyanate, 1 m ammonium thiocyanate, 0.1 m sodium acetate, 5% glycerol, 38% water saturated phenol), followed by chloroform extraction. RNA was precipitated in 0.5 × isopropanol for 20 min. Triplicate samples from pools of two leaves were analyzed.

#### Real‐time RT‐qPCR

A real‐time RT‐qPCR was performed essentially as described previously (López‐Dolz et al., 2020) in a QuantStudio 3 Real‐Time PCR System (Thermo Fisher Scientific, Waltham, MA, USA). *NbSu* target RNA expression levels were calculated relative to *N. benthamiana PROTEIN PHOSPHATASE 2A* (*NbPP2A*) reference gene using the delta delta cycle threshold comparative method of quantstudio design and analysis software, version 1.5.1 (Thermo Fisher Scientific). The primers used for RT‐qPCR are listed in Table S2.

### Small RNA blot assays

Total RNA (20 μg) was separated in 17% polyacrylamide gels containing 0.5× Tris/Borate EDTA and 7 m urea and transferred to positively charged nylon membrane. Probe synthesis using [γ‐^32^P]ATP (PerkinElmer, Waltham, MA, USA) and T4 polynucleotide kinase (Thermo Fisher Scientific) and northern‐blot hybridizations were performed at 38°C in PerfectHyb Plus Hybridization Buffer (Sigma‐Aldrich, St Louis, MO, USA) as described previously (Carbonell et al., 2014; Montgomery, Howell, et al., 2008)**.** A Typhoon IP Imager System (Cytiva, Marlborough, MA, USA) was used to produce digital images from radioactive membranes, and band quantification was done using imagequant tl, version 10.0 (Cytiva). The oligonucleotides used as probes for sRNA blots are listed in Table S2.

### Microscopy

Whole leaves were placed in a Petri dish, illuminated by two lateral light sources. Images were obtained under bright field with a Magnifier MZ16F stereomicroscope in conjunction with las, version 4.12 (Leica, Wetzlar, Germany). Sections of 1 cm^2^ were cut from whole leaves, placed between a glass slide and a coverslip and observed under a Leica 500 microscope using las, version 4.9 and a 40× objective (40×/0.75 HCX PL Fluotar; Leica). Images were obtained under bright field and under an UV‐A filter (340–380 nm excitation; 425 nm emission).

### 5′‐RLM‐race

RNA ligase‐mediated rapid amplification of 5′ cDNA ends was done using the GeneRacer kit (Life Technologies, Carlsbad, CA, USA) as described previously (Carbonell et al., 2015), except that the 5′ end of cDNA specific to *NbSu* was directly amplified in a single PCR using the GeneRacer 5′ and gene‐specific AC‐532 oligonucleotides. 5′‐RLM‐RACE products were gel purified and cloned using the Zero Blunt TOPO PCR Cloning Kit (Life Technologies), introduced in *E. coli* DH5α, screened for inserts and sequenced. Control PCR reactions to amplify *NbPP2A* were performed using oligonucleotides AC‐365 and AC‐366. The sequences of the oligonucleotides used are listed in Table S2.

### Small RNA sequencing and data analysis

Total RNA was analyzed for quantity, purity and integrity with a 2100 Bioanalyzer (RNA 6000 Nano kit; Agilent, Santa Clara, CA, USA) and submitted to BGI (Hong Kong, China) for sRNA library preparation and sRNA sequencing in a DNBSEQ Platform (MGI Tech Co., Ltd, Shenzen, China). After reception of quality‐trimmed, adaptor‐removed clean reads from BGI, fastx_collapser (Hannon, 2010) was used to collapse identical reads into a single sequence, at the same time as maintaining read counts. A custom python script (https://www.python.org) was then used to map each clean, unique read against the forward and reverse strands of both the Nbv5.1tr6204879 transcript (Data S1) and the precursor of the art‐sRNA overexpressed in each sample (Data S3), not allowing mismatches or gaps. The Python script was also used to calculate the counts and RPMs (RPM mapped reads) for each mapping position.

Processing accuracy of amiRNA foldbacks and syntasiRNA transcripts was assessed by quantifying the proportion of 19–24‐nucleotide sRNA (+) reads that mapped within ±4 nucleotides of the 5′ end of the amiRNA guide or DCL4 processing position 3′D2[+], respectively, as reported previously (Carbonell et al., 2015; Cuperus et al., 2010). Phasing register tables were built by calculating the proportion of 21‐nucleotide sRNA (+) reads in each register relative to the amiR‐NbSu‐2 cleavage site for all 21‐nucleotide positions downstream of the cleavage site, as described previously (Carbonell et al., 2014).

## ACCESSION NUMBERS

The *N. benthamiana* and *E. coli* genes and corresponding locus identifiers are *NbSu* (Nbv5.1tr6204879), *NbPP2A* (Nbv5.1tr6224808) and *EcGUS* (AJ571699). High‐throughput sequencing data can be found in the Sequence Read Archive (SRA) database under accession number PRJNA811353.

## CONFLICT OF INTEREST

The authors declare no conflict of interest.

## AUTHOR CONTRIBUTIONS

AC planned and designed the research. AEC, AdlT‐M and AC performed the experiments. AEC and AC analyzed data and AC wrote the manuscript.

## Supporting information


**Figure S1** **.** Genetic analysis of local and systemic silencing triggered by amiR‐NbSu‐2 in wild‐type and in DCL4 (DCL4i) or RDR6 (RDR6i) knockdown plants.
**Figure S2.** Bar graphs showing the number of amiR‐GUS_Nb_ and amiR‐NbSu‐2 reads in *35S:amiR‐GUS*_*Nb*_ and *35S:amiR‐NbSu‐2* expressing tissues and in upper leaves.
**Figure S3.** Effects on systemic silencing of the place of expression of syn‐tasiR‐NbSu‐2 in *N. benthamiana* leaves.
**Figure S4.** Speed of translocation of the systemic silencing (SS) signal.
**Figure S5.** Bar graphs showing the number of syn‐tasiR‐GUS_Nb_ and syn‐tasiR‐NbSu‐2 reads in *35S:syn‐tasiR‐GUS*_*Nb*_ and *35S:syn‐tasiR‐NbSu‐2* expressing tissues and in upper leaves.

**Table S1** **.** Artificial small RNA‐induced systemic silencing (SS) appearance as leaf near‐vein chlorosis in upper or lower non‐agroinfiltrated leaves during 14 days post‐agroinfiltration.
**Table S2.** Name, sequence and use of DNA oligonucleotides used in the present study.

**Data S1** **.** *NbSu*‐derived sRNA reads from amiRNA‐ and syn‐tasiRNA‐expressing tissues and from upper leaves.

**Data S2** **.** Complete list of optimal results generated by P‐SAMS amiRNA Designer for the designs of amiRNAs against NbSu and GUS with no off‐targets in *N. benthamiana*.

**Data S3** **.** sRNA reads from amiRNA‐ and syn‐tasiRNA‐expressing tissues and from upper leaves.

## Data Availability

All data relating to this manuscript can be found within the manuscript and its supplementary files. Data that support the findings of this study are available from the corresponding author upon reasonable request. High‐throughput sequencing data can be found in the Sequence Read Archive (SRA) database under accession number PRJNA811353.
