# Supplementary material for: Systemic silencing of an endogenous plant gene by two classes of mobile 21‐nucleotide artificial small RNAs
Source: Plant J. 2022 Mar 27;110(4):1166–81. doi: 10.1111/tpj.15730 (PMC9310713; doi:10.1111/tpj.15730)
Supplement: Supplementary file 1 — Figure S1 . Genetic analysis of local and systemic silencing triggered by amiR‐NbSu‐2 in wild‐type and in DCL4 (DCL4i) or RDR6 (RDR6i) knockdown plants. Figure S2. Bar graphs showing the number of amiR‐GUSNb and amiR‐NbSu‐2 reads in 35S:amiR‐GUS Nb and 35S:amiR‐NbSu‐2 expressing tissues and in upper leaves. Figure S3. Effects on systemic silencing of the place of expression of syn‐tasiR‐NbSu‐2 in N. benthamiana leaves. Figure S4. Speed of translocation of the systemic silencing (SS) signal. Figure S5. Bar graphs showing the number of syn‐tasiR‐GUSNb and syn‐tasiR‐NbSu‐2 reads in 35S:syn‐tasiR‐GUS Nb and 35S:syn‐tasiR‐NbSu‐2 expressing tissues and in upper leaves. [file TPJ-110-1166-s003.pdf]

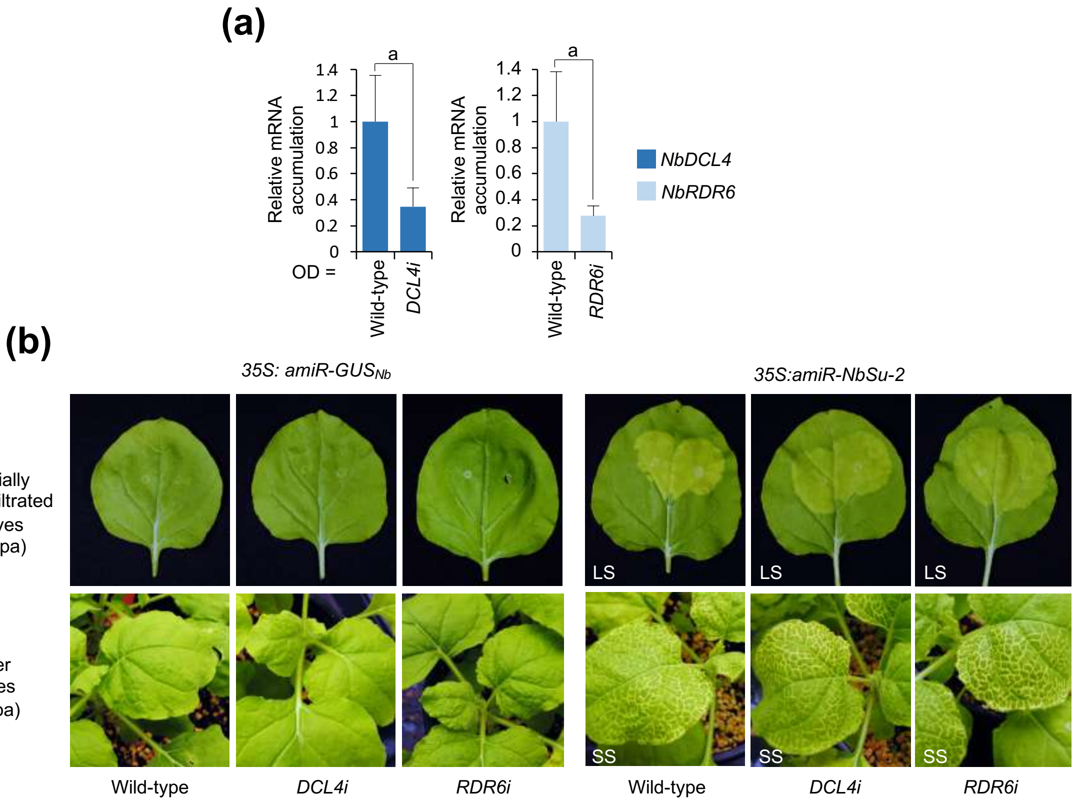

Fig. S1. Genetic analysis of local and systemic silencing triggered by amiR-NbSu-2 in wild-type and in DCL4 (DCL4i) or RDR6 (RDR6i) knockdown plants. (a) Accumulation of *NbDCL4* and *NbRDR6* mRNAs in leaves of wilt-type, *DCL4i* and *RDR6i N. benthamiana* plants. Mean relative level (n=3) + standard error of mRNAs after normalization to *PROTEIN PHOSPHATASE 2A* (*PP2A*), as determined by quantitative RT-PCR (qPCR) (Wild-type = 1.0 in all comparisons). Other details are as in Fig 1b. (b) Photos of partially agroinfiltrated leaves at 7 days-post agroinfiltration (dpa) (top), and of upper non-agroinfiltrated leaves at 14 dpa (bottom). Other details are as in Fig. 2a.

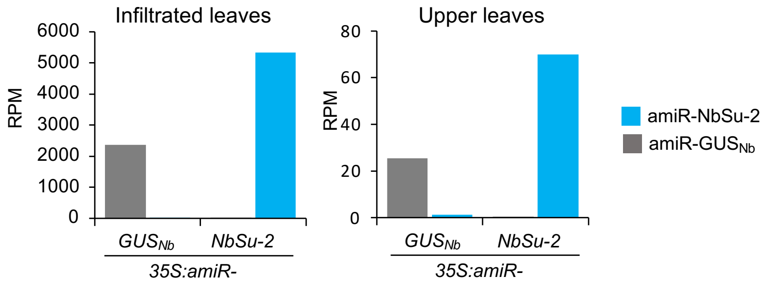

Fig. S2. Bar graphs showing the number of amiR-GUS$_{\mathrm{Nb}}$ and amiR-NbSu-2 reads in *35S:amiR-GUS*$_{Nb}$ and *35S:amiR-NbSu-2* expressing tissues and in upper leaves.

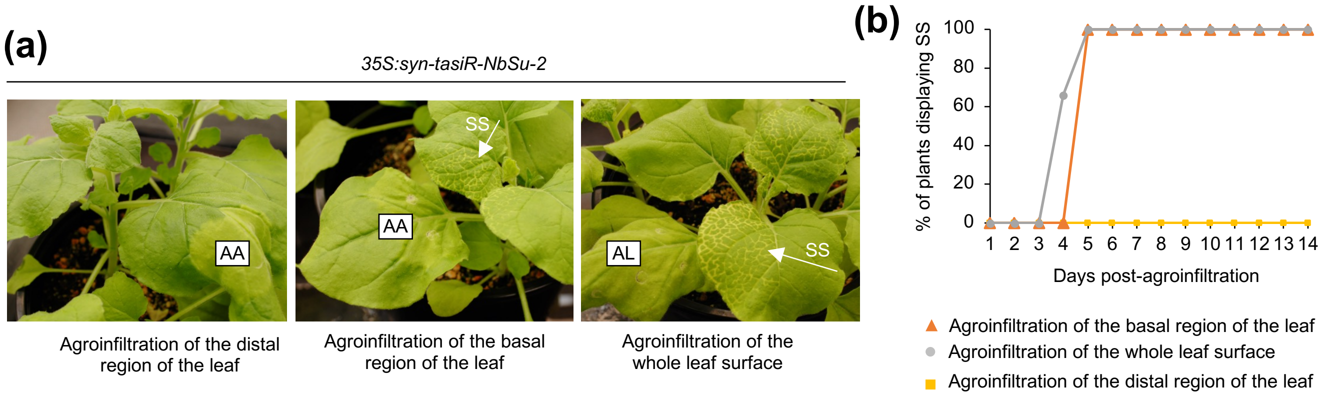

Fig. S3. Effects on systemic silencing of the place of expression of syn-tasiR-NbSu-2 in *N. benthamiana* leaves. (a) Photos of plants at 7 days post-agroinfiltration (dpa). AA and AL refer to agroinfiltrated area and agroinfiltrated leaf, respectively. Near-vein chlorotic areas result of systemic silencing (SS) are pointed with a white arrow. (b) Two-dimensional line graph showing, for each of the three-plant sets listed in the box, the percentage of plants displaying systemic silencing per day during 14 dpa.

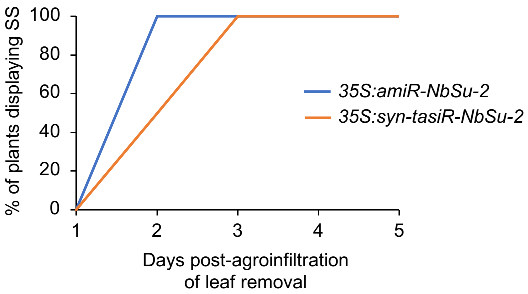

Fig. S4. Speed of translocation of the systemic silencing (SS) signal. Bar graph shows the percentage of plants displaying SS after removal of the agroinfiltrated leaves at 1, 2, 3, 4 or 5 days post-agroinfiltration (dpa).

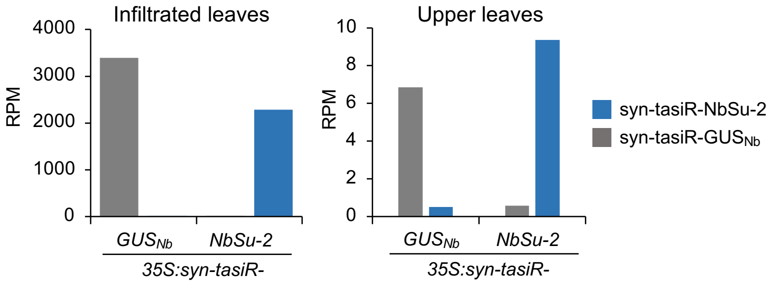

Fig. S5. Bar graphs showing the number of syn-tasiR-GUS$_{Nb}$ and syn-tasiR-NbSu-2 reads in *35S:syn-tasiR-GUS$_{Nb}$* and *35S:syn-tasiR-NbSu-2* expressing tissues and in upper leaves.
