# Supplementary material for: Systemic silencing of an endogenous plant gene by two classes of mobile 21‐nucleotide artificial small RNAs
Source: Plant J. 2022 Mar 27;110(4):1166–81. doi: 10.1111/tpj.15730 (PMC9310713; doi:10.1111/tpj.15730)
Supplement: Supplementary file 2 — Table S1 . Artificial small RNA‐induced systemic silencing (SS) appearance as leaf near‐vein chlorosis in upper or lower non‐agroinfiltrated leaves during 14 days post‐agroinfiltration. Table S2. Name, sequence and use of DNA oligonucleotides used in the present study. [file TPJ-110-1166-s004.pdf]

Table S1. Artificial small RNA-induced systemic silencing (SS) appearance as leaf near-vein chlorosis in upper or lower non-agroinfiltrated leaves during 14 days post-agroinfiltration.

Art-sRNA construct	Agroinfiltrated leaves*	SS in lower leaves	SS in upper leaves
35S:amiR-NbSu-2	3-4	0/3	3/3
	5-6	0/3	3/3
	7-8	0/3	3/3
35S:syn-tasiR-NbSu-2	3-4	0/3	3/3
	5-6	0/3	3/3
	7-8	0/3	3/3

*leaf numbers refer to the age of the leaf, with leaf number 1 being the oldest leaf

Table S2. Name, sequence and use of DNA oligonucleotides used in this study.

**Oligonucleotide**	**Sequence**	**Construct/Aim**
AC-55	AGGGGCCATGCTAATCTTCTC	DNA probe for U6 detection
AC-257	TTTATGTATGACTCCCGGAATTCCA	*35S:syn-tasiR-NbSu-2*
AC-258	CCGATGGAATTCCGGGAGTCATACA	
AC-310	TGTATGTATGACTCCCGGAATTCCTTATGATGATCACA TTCGTTATCTATTTTTTAAGGATTCCGTGAGTCATACA	*35S:amiR-NbSu-2-22*
AC-311	AATGTGTATGACTCACGGAATCCTTAAAAAATAGATAA CGAATGTGATCATCATAAGGAATTCCGGGAGTCATACA	
AC-337	TATAGGGGGGAAAAAAAGGTAG	PCR *AtMIR390a*-based amiRNA precursors
AC-338	GAGACTAAAGATGAGATCTAAT	
AC-346	T+GGA+ATT+CCG+GGA+GTC+ATA+CA	LNA probe for amiR-NbSu-2/syn-tasiR-NbSu-2 detection
AC-355	TCACACCCTGCCCGATTTAT	*NbSu* qPCR
AC-356	GCAGAAAGAGCGTTCCTAGC	
AC-365	GACCCTGATGTTGATGTTCGCT	*NbPP2A* PCR/qPCR
AC-366	GAGGGATTTGAAGAGAGATTTC	
AC-417	G+CGG+GAA+GTC+CAC+CAC+GGT+TA	LNA probe for amiR-NbSu-1 detection
AC-419	T+AGA+TGG+TTG+ATT+TAC+CGC+TA	LNA probe for amiR-NbSu-3 detection
AC-532	CTCGTGCTGCCCTGTTAGTGACTATA	*NbSu* PCR (5'RLM-RACE)
D2057	TGTATCTTGTAACGCGCTTTCCCAGATGATGATCACAT TCGTTATCTATTTTTTCTGGGAAAGCTCGTTACAAGA	*35S:amiR-GUS*$_{Nb}$
D2058	AATGTCTTGTAACGAGCTTTCCCAGAAAAAATAGATAA CGAATGTGATCATCATCTGGGAAAGCGCGTTACAAGA	
D2065	TGTATAACCGTGGTGGACTTCCCGCATGATGATCACAT TCGTTATCTATTTTTTGCGGGAAGTCAACCACGGTTA	*35S:amiR-NbSu-1*
D2066	AATGTAACCGTGGTTGACTTCCCGCAAAAAATAGATAA CGAATGTGATCATCATGCGGGAAGTCCACCACGGTTA	
D2067	TGTATGTATGACTCCCGGAATTCCAATGATGATCACAT TCGTTATCTATTTTTTTGGAATTCCGTGAGTCATACA	*35S:amiR-NbSu-2*
D2068	AATGTGTATGACTCACGGAATTCCAAAAAAATAGATAA CGAATGTGATCATCATTGGAATTCCGGGAGTCATACA	
D2224	AAACCTAAACCTAAACGGCTAAGC	PCR *AtTAS1c*-based syn-tasiRNA precursors
D2225	ATTTCACTTTACGATGTGGTG	
GeneRacer 5' Oligo	CGACTGGAGCACGAGGACACTGA	*NbSu* PCR (5'RLM-RACE)
GeneRacer Oligo dT	GCTGTCAACGATACGCTACGTAACGGCATGACAGTGTT TTTTTTTTTTTTTTTTTTTTTTTT	cDNA synthesis (5'RLM-RACE)
GeneRacer RNA Oligo Adapter	CGACUGGAGCACGAGGACACUGACAUGGACUGAAGGAG UAGAAA	RNA ligation (5'RLM-RACE)
